# Risk factors associated with revision for prosthetic joint infection after hip replacement: a prospective observational cohort study

**DOI:** 10.1016/S1473-3099(18)30345-1

**Published:** 2018-09

**Authors:** Erik Lenguerrand, Michael R Whitehouse, Andrew D Beswick, Setor K Kunutsor, Ben Burston, Martyn Porter, Ashley W Blom

**Affiliations:** a Musculoskeletal Research Unit, Translational Health Sciences, Bristol Medical School, University of Bristol, Bristol, UK; b National Institute for Health Research Bristol Biomedical Research Centre, University Hospitals Bristol National Health Service (NHS) Foundation Trust, and University of Bristol, Bristol, UK; c The Robert Jones and Agnes Hunt Orthopaedic Hospital, Oswestry, UK; d Centre for Hip Surgery, Wrightington Hospital, Wrightington, Wigan and Leigh NHS Trust, Lancashire, UK

## Abstract

**Background:**

The risk of prosthetic joint infection (PJI) is influenced by patient, surgical, and health-care factors. Existing evidence is based on short-term follow-up. It does not differentiate between factors associated with early onset caused by the primary intervention from those associated with later onset more likely to result from haematogenous spread. We aimed to assess the overall and time-specific associations of these factors with the risk of revision due to PJI after primary total hip replacement.

**Methods:**

We did a prospective observational cohort study analysing 623 253 primary hip procedures performed between April 1, 2003, and Dec 31, 2013, in England and Wales and recorded the number of procedures revised because of PJI. We investigated the associations between risk factors and risk of revision for PJI across the overall follow-up period using Poisson multilevel models. We reinvestigated the associations by post-operative time periods (0–3 months, 3–6 months, 6–12 months, 12–24 months, >24 months) using piece-wise exponential multilevel models with period-specific effects. Data were obtained from the National Joint Registry linked to the Hospital Episode Statistics data.

**Findings:**

2705 primary procedures were subsequently revised for an indication of PJI between 2003 and 2014, after a median (IQR) follow up of 4·6 years (2·6–7·0). Among the factors associated with an increased revision due to PJI there were male sex (1462 [1·2‰] of 1 237 170 male-years *vs* 1243 [0·7‰] of 1 849 691 female-years; rate ratio [RR] 1·7 [95% CI 1·6–1·8]), younger age (739 [1·1‰] of 688 000 person-years <60 years *vs* 242 [0·6‰] of 387 049 person-years ≥80 years; 0·7 [0·6–0·8]), elevated body-mass index (BMI; 941 [1·8‰] 517 278 person-years with a BMI ≥30 kg/m^2^*vs* 272 [0·9‰] of 297 686 person-years with a BMI <25 kg/m^2^; 1·9 [1·7–2·2]), diabetes (245 [1·4‰] 178 381 person-years with diabetes *vs* 2120 [1·0‰] of 2 209 507 person-years without diabetes; 1·4 [1·2–1·5]), dementia (5 [10·1‰] of 497 person-years with dementia at 3 months *vs* 311 [2·6‰] of 120 850 person-years without dementia; 3·8 [1·2–7·8]), previous septic arthritis (22 [7·2‰] of 3055 person-years with previous infection *vs* 2683 [0·9‰] of 3 083 806 person-years without previous infection; 6·7 [4·2–9·8]), fractured neck of femur (66 [1·5‰] of 43 378 person-years operated for a fractured neck of femur *vs* 2639 [0·9‰] of 3 043 483 person-years without a fractured neck of femur; 1·8 [1·4–2·3]); and use of the lateral surgical approach (1334 [1·0‰] of 1 399 287 person-years for lateral *vs* 1242 [0·8 ‰] of 1 565 913 person-years for posterior; 1·3 [1·2–1·4]). Use of ceramic rather than metal bearings was associated with a decreased risk of revision for PJI (94 [0·4‰] of 239 512 person-years with ceramic-on-ceramic bearings *vs* 602 [0·5‰] of 1 114 239 peron-years with metal-on-polyethylene bearings at ≥24 months; RR 0·6 [0·4–0·7]; and 82 [0·4‰] of 190 884 person-years with ceramic-on-polyethyene bearings *vs* metal-on-polyethylene bearings at ≥24 months; 0·7 [0·5–0·9]). Most of these factors had time-specific effects. The risk of revision for PJI was marginally or not influenced by the grade of the operating surgeon, the absence of a consultant surgeon during surgey, and the volume of procedures performed by hospital or surgeon.

**Interpretation:**

Several modifiable and non-modifiable factors are associated with the risk of revision for PJI after primary hip replacement. Identification of modifiable factors, use of targeted interventions, and beneficial modulation of some of these factors could be effective in reducing the incidence of PJI. It is important for clinicians to consider non-modifiable factors and factors that exhibit time-specific effects on the risk of PJI to counsel patients appropriately preoperatively.

**Funding:**

National Institute for Health Research.

## Introduction

Hip replacement is a successful and cost-effective elective surgical intervention that is widely used to treat disabling joint pain, mainly caused by osteoarthritis. Some patients experience complications and one of the most severe is prosthetic joint infection (PJI),^1^ which is most commonly caused by coagulase-negative staphylococcus or *Staphylococcus aureus*.^2^ Although uncommon, PJI is devastating and leads to severe pain, poor function, reduced quality of life, and even death.^1^, ^3^ The treatment burden is high for patients and health-care systems.^4^ Revision surgery is usually required and is complex, protracted, and associated with further complications.^5^, ^6^ A large rise in the number of primary hip replacements is predicted^7^ and a proportionate rise in the number of patients requiring revision for PJI is expected.^8^ In England and Wales alone, over 1000 revision procedures are performed annually because of PJI of the hip.^9^

**Research in context**

**Evidence before this study**

Prosthetic joint infection (PJI) is a devastating complication after hip replacement. In a systematic review published in 2016, we searched MEDLINE, Embase, Web of Science, and The Cochrane Library databases from inception up to Sept 1, 2015, using a registered protocol (PROSPERO: CRD42015023485) to identify the role of patient characteristics on the risk of developing PJI. Our search strategy combined terms related to exposures (eg, “risk factor”, “body mass index”, “comorbidity”) with those related to outcomes (eg, “periprosthetic joint infection”, “prosthetic joint infection”, “deep prosthetic infection”, “deep infection”, “deep surgical site infection”). Longitudinal studies that reported on the associations of any patient factors with PJI after primary or revision total arthroplasty, who had at least 12 months of follow-up and who had a Newcastle-Ottawa Scale score of more than 5 were eligible. 512 508 hip replacements were pooled and showed that male sex, high body-mass index (BMI), steroid use, diabetes, rheumatoid arthritis, congestive heart failure, depression, and smoking and alcohol intake are each associated with an increased risk of PJI. The published literature was limited by short-term postoperative follow-up, variably adjusted data which did not enhance consistent comparison, substantial heterogeneity between contributing studies, and by not disentangling factors associated with early onset of PJI caused by the primary intervention from factors associated with later onset resulting from haematogenous spread. Older reviews had investigated the role of surgical intervention and health-care setting factors on the risk of revision for PJI but were also limited by the size of the studied samples, infected cases, short postoperative follow-up (≤12 months), and between study heterogeneity.

Repeating the search on March 19, 2018, we identified two registry studies and a meta-analysis published since our previous review. Registry studies from Denmark and New Zealand observed increased risk of PJI in men, older patients, those with a high BMI, and those with rheumatoid arthritis. The meta-analysis showed weak evidence of reduced risk of PJI for non-metallic bearing surfaces. The authors highlighted the need for larger studies with adjustment for confounders.

**Added value of this study**

This study investigated the overall and postoperative period-specific effects of patient, surgical, and health system factors on the risk of revision for PJI, with a single dataset of 623 053 primary hip replacements in which patients were followed up for up to 11 years. Considering patient characteristics, this work corroborates the previous findings of our review and identifies other factors such as younger age, chronic pulmonary disease, liver disease, and dementia that are associated with an increased risk of PJI. Surgical factors, including indication for the primary surgery, surgery type, the lateral surgical approach, and non-ceramic bearing surfaces, were associated with an increased risk of PJI. We identified no effects or only small effects for surgeon and hospital volume or surgeon grade. More importantly, we identified that these factors have a different effect according to the postoperative period considered, with comorbidities such as dementia influencing early revision for PJI and liver diseases influencing long-term revision. The effect of bearing surfaces also varied according to the period considered but factors, such as age or BMI, increased the risk during all postoperative periods.

**Implications of all the available evidence**

The risk of revision for PJI after primary hip replacement is multifactorial, mainly driven by patient and surgical level factors with time-varying effects. The modifiable factors identified in this study should be considered by clinicians in their practice to develop targeted interventions and propose beneficial modulation of some of these factors. Of equal importance is for clinicians to consider the non-modifiable factors and the factors that exhibit time-specific effects on the risk of PJI, to counsel patients appropriately preoperatively.

Identification of individuals at high risk of PJI helps to inform the development of preventive strategies and optimise the detection of PJI. The risk of developing PJI is influenced by non-modifiable and modifiable patient, surgical, and health-care characteristics. In our systematic review^10^ of patient risk factors for PJI, we identified male sex, smoking, increasing body-mass index (BMI), steroid use, previous joint surgery, and comorbidities, such as diabetes, rheumatoid arthritis, and depression. Limitations of this review included short-term follow-up, pooled estimates based on variably adjusted data, and evidence of substantial heterogeneity between study settings. These limitations are also applicable to other systematic reviews of surgical and health-care system factors associated with revision for PJI.^11^, ^12^

Given these limitations, large-scale cohort studies are needed with adequate power to provide evidence on the nature and magnitude of the associations of potential risk factors for PJI. It is important to disentangle factors associated with early onset of PJI, which are likely to be the consequence of the primary intervention, from factors associated with later onset, which are more likely to result from haematogenous spread.^6^

We aimed to assess the overall and postoperative period-specific associations of patient, surgical, and health-care setting factors with the risk of revision due to PJI in prospectively collected observational data of 623 253 primary total hip replacements performed in England and Wales.

## Methods

### Study design and participants

In this observational cohort study, we report analyses of data for England and Wales from the National Joint Registry (NJR) for England, Wales, Northern Ireland, and the Isle of Man between April 1, 2003, and Dec 31, 2014.

NJR data were linked to Hospital Episode Statistics and Patient Episode Database for Wales to obtain data on inpatient and day case admissions. Data from the Office for National Statistics were linked to obtain the date of death.

We included all patients who had a primary hip replacement between April 1, 2003, and Dec 31, 2013, in the study. Patient consent was obtained for data collection and linkage by the NJR. According to the National Health Service Health Research Authority, separate consent and ethical approval were not required for this study.

### Procedures

We analysed primary hip replacements performed between April 1, 2003, and Dec 31, 2013, and revision procedures due to PJI that occurred after the primary replacement between April 1, 2003, and Dec 31, 2014. The reason for revision was recorded by clinicians at the time of the revision procedure and reflected a clinical judgment sufficient to lead the surgeon to perform an invasive procedure tailored to tackle a PJI. The diagnosis and treatment strategy for PJI was at the discretion of the surgeon and treating unit and was reflective of contemporary practice over the study period, with raised inflammatory markers, joint specific symptoms, sinuses, and positive microbiological cultures^13^ being common diagnostic features over that period.

Each patient who had a primary hip replacement was followed up for a minimum of 12 months until the end of the observation period (Dec 31, 2014) or until the date of revision for PJI, revision for another indication, or death. Revisions for PJI included debridement and implant retention with modular exchange, a single or a two-stage revision procedure.^14^

We considered the patient characteristics age, sex, ethnicity, BMI, American Society of Anaesthesiologists (ASA) grade, and comorbidities. We obtained data for ethnicity and comorbidities from the Hospital Episode Statistics records. We used ICD-10 codes to classify comorbidities for which patients had been admitted to hospital in the 5 years preceding their primary operation (appendix).^15^

We considered surgical factors such as indication for surgery, anaesthesia type, thromboprophylaxis regime, surgical approach, hip replacement type, bearing surface, use of bone graft, and occurrence of intraoperative complications.

We considered health system factors such as hospital type, funding stream, country, operating surgeon grade, consultant involvement, and volume of hip surgeries (categorised into quartiles) performed by the hospital, operating surgeon and surgeon in charge of the procedure in the preceding 12 months.

### Statistical methods

We first investigated the associations between the risk factors and risk of revision for PJI across the overall follow-up period. We used Poisson multilevel models accounting for clustering at unit level (random intercept). Clustering at surgeon level was negligible and therefore ignored.

PJI management varies according to the time since the primary procedure and onset of infection. Early onset of PJI within 24 months of primary procedure is generally considered to result from the primary intervention. Later onset of PJI is more likely to be due to haematogenous spread. For patients with early post-operative or acute haematogenous PJI and a short duration of symptoms, debridement, modular exchange, and implant retention rather than full revision are appropriate.^6^ Therefore, we reinvestigated the associations over several at-risk postoperative periods: 0–3 months, 3–6 months, 6–12 months, 12–24 months, and more than 24 months. We split each patient's at-risk period (time elapsed between their primary procedure and endpoint) according to the time spent in each of these periods and we assigned the revision for PJI status (revised for PJI or not) to the relevant period. We used a piece-wise exponential multilevel model with period-specific effects to assess these associations—ie, their rate ratios (RR) and 95% CIs across these time-periods.^16^, ^17^ We did analyses by running MLwiN from Stata 14.1 (StataCorp LP, TX, USA) using Markov Chain Monte Carlo methods.^18^ To account for test multiplicity, we derived adjusted p values using Simes' false discovery rate testing controlling procedure.^19^, ^20^ To be confident that 95% of the effects tested were not due to chance, we only discussed evidence of association for adjusted p value of 0·05 or lower.

We did the analyses on the overall sample for all exposures except for ethnicity and comorbidities, which we investigated in the 495 456 patients operated on in England with a record of hospital admission in HES but not in PEDW, and no evidence of residency outside England (appendix). We adjusted the regressions for age, sex, ASA grade, and BMI. BMI is an important risk factor for PJI but has substantial missing data in the NJR (47%), partly because it was not included as a variable in the early data collection forms. We used a multiple imputation strategy to impute BMI, assuming that data were missing at random, using a Gaussian normal regression imputation model with the factors age, sex, and ASA used as covariates, and the log of the observed event or censoring time and revision for PJI status. Due to the computational time required by each multilevel piece-wise model, we computed only five imputations and combined regression estimates by Rubin's rules. Unadjusted and adjusted models without BMI are available on request. To avoid overadjustment, we did not adjust models investigating the effect of comorbidities for ASA grade, a proxy indicator of comorbid profile.

### Role of the funding source

The National Institute for Health Research had no role in study design, data collection analysis, interpretation, or writing of the report. The corresponding author had full access to all the data in the study and had final responsibility for the decision to submit for publication.

## Results

**Figure 1:**
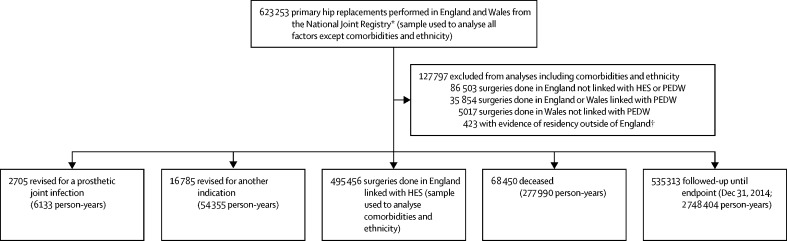
Description of the sample HES=Hospital Episode Statistics for England. PEDW=Patient Episode Database for Wales. *Only data for England and Wales were considered; data collection for Northern Ireland started Feb 2, 2013, and primary revision procedures could not be considered due to their limited number and restricted follow-up. Data collection for the Isle of Man started on July 1, 2015, after the endpoint of this study and were not considered. †As recorded in HES for the 5 years preceding the primary hip replacement.

623 253 primary hip procedures were done in 460 different surgical units with a median (IQR) of 1050 (460–1940) per unit. Baseline study sample characteristics are presented in figure 1 and the table. 2705 primary procedures were subsequently revised for an indication of PJI after a median (IQR) follow-up of 4·6 years (2·6–7·0); 14% (n=372) of these within 3 months, 8% (n=204) in 3–6 months, 14% (n=374) in 6–12 months, 23% (n=612) in 12–24 months, and 42% (n=1143) beyond 24 months from the primary procedure. The mean patient age was 68 years (SD 11). The sample is presented by time periods in the appendix. In 523 (27%) of the 1959 two-stage revision procedures performed for PJI, only a second stage procedure was recorded in the NJR. Patients with incompletely registered two-stage procedures did not differ from those with complete procedures and their time to first-stage procedure was estimated (appendix).

**Table:** Sample description and incidence rates

	**Patients, n**	**Person-years**	**Cases, n**	**Incidence per 1000 person-years (95% CI)**
**Sex**				
Female	372 256	1 849 691	1243	0·67 (0·64–0·71)
Male	250 997	1 237 170	1462	1·18 (1·12–1·24)
**Age, years**				
<60	131 803	688 000	739	1·07 (1·00–1·15)
60–69	191 128	977 963	942	0·96 (0·90–1·03)
70–79	210 387	1 033 850	782	0·76 (0·70–0·81)
≥80	89 935	387 049	242	0·63 (0·55–0·71)
**Ethnicity**				
White	469 129	2 256 675	2308	1·02 (0·98–1·07)
Black African origin	2855	13 152	12	0·91 (0·47–1·59)
South Asian	1605	7223	6	0·83 (0·30–1·81)
Other and mixed	3235	14 405	14	0·97 (0·53–1·63)
Unclear	18 632	96 433	25	0·26 (0·17–0·38)
Unavailable*	127 797	698 972	340	0·49 (0·44–0·54)
**Body-mass index, kg/m^2^**				
<25	71 584	297 686	272	0·91 (0·81–1·03)
25–29·9	133 037	557 826	580	1·04 (0·96–1·13)
≥30	125 856	517 278	941	1·82 (1·70–1·94)
Missing	292 776	1 714 072	912	0·53 (0·50–0·57)
**American Society of Anaesthesiologists grade**				
1	114 367	657 059	482	0·73 (0·67–0·80)
2	418 335	2 036 022	1772	0·87 (0·83–0·91)
3–5	90 551	393 780	451	1·15 (1·04–1·26)
**Chronic pulmonary disease**				
No	433 003	2 127 270	2064	0·97 (0·93–1·01)
Yes	62 453	260 618	301	1·15 (1·03–1·29)
Unavailable*	127 797	698 972	340	0·49 (0·44–0·54)
**Diabetes**				
No	453 057	2 209 507	2120	0·96 (0·92–1·00)
Yes	42 399	178 381	245	1·37 (1·21–1·56)
Unavailable*	127 797	698 972	340	0·49 (0·44–0·54)
**Dementia**				
No	493 382	2 381 198	2355	0·99 (0·95–1·03)
Yes	2074	6690	10	1·49 (0·72–2·75)
Unavailable*	127 797	698 972	340	0·49 (0·44–0·54)
**Liver disease**				
No	491 430	2 372 883	2327	0·98 (0·94–1·02)
Yes	4026	15 005	38	2·53 (1·79–3·48)
Unavailable*	127 797	698 972	340	0·49 (0·44–0·54)
**Congestive heart failure**				
No	484 748	2 346 960	2307	0·98 (0·94–1·02)
Yes	10 708	40 928	58	1·42 (1·08–1·83)
Unavailable*	127 797	698 972	340	0·49 (0·44–0·54)
**Connective tissue and rheumatic disease**				
No	473 594	2 292 733	2251	0·98 (0·94–1·02)
Yes	21 862	95 156	114	1·20 (0·99–1·44)
Unavailable*	127 797	698 972	340	0·49 (0·44–0·54)
**Cancer**				
No	473 046	2 299 171	2262	0·98 (0·94–1·03)
Non-metastatic cancer	18 511	77 688	85	1·09 (0·87–1·35)
Metastatic cancer	3899	11 030	18	1·63 (0·97–2·58)
Unavailable*	127 797	698 972	340	0·49 (0·44–0·54)
**Cerebrovascular disease**				
No	485 508	2 348 220	2329	0·99 (0·95–1·03)
Yes	9948	39 668	36	0·91 (0·64–1·26)
Unavailable*	127 797	698 972	340	0·49 (0·44–0·54)
**Myocardial infarction**				
No	481 922	2 330 894	2305	0·99 (0·95–1·03)
Yes	13 534	56 995	60	1·05 (0·80–1·36)
Unavailable*	127 797	698 972	340	0·49 (0·44–0·54)
**Paraplegia and hemiplegia**				
No	493 415	2 379 416	2351	0·99 (0·95–1·03)
Yes	2041	8472	14	1·65 (0·90–2·77)
Unavailable*	127 797	698 972	340	0·49 (0·44–0·54)
**Peptic ulcer disease**				
No	488 994	2 358 642	2333	0·99 (0·95–1·03)
Yes	6462	29 247	32	1·09 (0·75–1·54)
Unavailable*	127 797	698 972	340	0·49 (0·44–0·54)
**Peripheral vascular disease**				
No	485 720	2 349 624	2318	0·99 (0·95–1·03)
Yes	9736	38 265	47	1·23 (0·90–1·63)
Unavailable*	127 797	698 972	340	0·49 (0·44–0·54)
**Renal disease**				
No	479 616	2 337 545	2311	0·99 (0·95–1·03)
Yes	15 840	50 343	54	1·07 (0·81–1·40)
Unavailable*	127 797	698 972	340	0·49 (0·44–0·54)
**Osteoarthritis**				
No	43 673	189 279	249	1·32 (1·16–1·49)
Yes	579 580	2 897 582	2456	0·85 (0·81–0·88)
**Fractured neck of femur**				
No	610 693	3 043 483	2639	0·87 (0·83–0·90)
Yes	12 560	43 378	66	1·52 (1·18–1·94)
**Previous hip infection**				
No	622 597	3 083 806	2683	0·87 (0·84–0·90)
Yes	656	3055	22	7·20 (4·51–10·90)
**Avascular necrosis**				
No	607 308	3 007 214	2597	0·86 (0·83–0·90)
Yes	15 945	79 647	108	1·36 (1·11–1·64)
**Dysplasia or congenital dislocation**				
No	613 710	3 038 036	2677	0·88 (0·85–0·92)
Yes	9543	48 825	28	0·57 (0·38–0·83)
**Inflammatory arthropathy**				
No	614 117	3 040 372	2665	0·88 (0·84–0·91)
Yes	9136	46 489	40	0·86 (0·61–1·17)
**Surgical approach**				
Posterior	337 188	1 565 913	1242	0·79 (0·75–0·84)
Lateral	257 487	1 399 287	1334	0·95 (0·90–1·01)
Other	28 578	121 661	129	1·06 (0·89–1·26)
**Procedure**				
Resurfacing	36 503	245 085	174	0·71 (0·61–0·82)
Total hip replacement cemented	229 008	1 196 702	1014	0·85 (0·80–0·90)
Total hip replacement uncemented	241 278	1 104 196	1074	0·97 (0·92–1·03)
Total hip replacement other	116 464	540 878	443	0·82 (0·74–0·90)
**Type of bearing**				
Metal-on-polyethylene	367 226	1 805 843	1505	0·83 (0·79–0·88)
Metal-on-metal	68 761	447 609	526	1·18 (1·08–1·28)
Ceramic-on-polyethylene	73 607	328 183	252	0·77 (0·68–0·87)
Ceramic-on-ceramic	99 651	428 600	342	0·80 (0·72–0·89)
Ceramic-on-metal or metal-on-ceramic	2263	10 553	20	1·90 (1·16–2·93)
Other	11 745	66 073	60	0·91 (0·69–1·17)
**General anaesthesia**				
No	323 710	1 532 200	1317	0·86 (0·81–0·91)
Yes	299 543	1 554 661	1388	0·89 (0·85–0·94)
**Nerve block anaesthesia**				
No	558 990	2 751 591	2426	0·88 (0·85–0·92)
Yes	64 263	335 270	279	0·83 (0·74–0·94)
**Epidural anaesthesia**				
No	568 425	2 752 938	2415	0·88 (0·84–0·91)
Yes	54 828	333 923	290	0·87 (0·77–0·97)
**Spinal anaesthesia used**				
No	244 716	1 302 912	1180	0·91 (0·85–0·96)
Yes	378 537	1 783 949	1525	0·85 (0·81–0·90)
**Thromboprophylaxis regimen**				
Chemical	562 884	2 691 005	2363	0·88 (0·84–0·91)
Non-chemical	60 369	395 856	342	0·86 (0·77–0·96)
**Acetabulum bonegraft**				
No	597 493	2 958 905	2588	0·87 (0·84–0·91)
Yes	25 760	127 956	117	0·91 (0·76–1·10)
**Femur bonegraft**				
No	618 407	3 062 174	2667	0·87 (0·84–0·90)
Yes	4846	24 687	38	1·54 (1·09–2·11)
**Intraoperative event**				
No	615 874	3 053 562	2663	0·87 (0·84–0·91)
Yes	7379	33 299	42	1·26 (0·91–1·70)
**Place of surgery**				
England	588 086	2 914 439	2539	0·87 (0·84–0·91)
Wales	35 167	172 422	166	0·96 (0·82–1·12)
**Funding**				
NHS	506 727	2 393 636	2172	0·91 (0·87–0·95)
Independent	90 650	500 738	354	0·71 (0·64–0·78)
Unspecified	25 876	192 487	179	0·93 (0·80–1·08)
**Grade of operating surgeon**				
Consultant	526 789	2 599 225	2253	0·87 (0·83–0·90)
Other	48 598	253 948	215	0·85 (0·74–0·97)
**Consultant involvement**				
Operating	526 789	2 599 225	2253	0·87 (0·83–0·90)
Assisting	33 262	158 323	163	1·03 (0·88–1·20)
Not involved	63 202	329 312	289	0·88 (0·78–0·98)
**Total volume (operating surgeon) of hip replacements performed in previous 12 months**				
≤28	164 527	928 504	836	0·90 (0·84–0·96)
29–63	158 385	797 348	752	0·94 (0·88–1·01)
64–148	153 734	718 359	583	0·81 (0·75–0·88)
>148	146 607	642 649	534	0·83 (0·76–0·90)
**Total volume (surgeon in charge) of hip replacements performed in previous 12 months**				
≤41	165 921	949 331	828	0·87 (0·81–0·93)
42–84	158 134	780 769	682	0·87 (0·81–0·94)
85–148	152 186	717 523	611	0·85 (0·79–0·92)
>148	147 012	639 238	584	0·91 (0·84–0·99)
**Total volume (hospital) of hip replacements performed in previous 12 months**				
≤143	160 375	960 028	789	0·82 (0·77–0·88)
144–256	158 020	799 114	659	0·82 (0·76–0·89)
257–406	154 586	671 995	581	0·86 (0·80–0·94)
>406	150 272	655 724	676	1·03 (0·95–1·11)

* Information on ethnicity and comorbidities are only available on the 495 456 patients operated in England with a Hospital Episode Statistics record—with no record in Patient Episode Database for Wales and no evidence of residency outside England (see figure 1 and appendix for more details).

RR of PJI revision surgery are presented in appendix.

**Figure 2:**
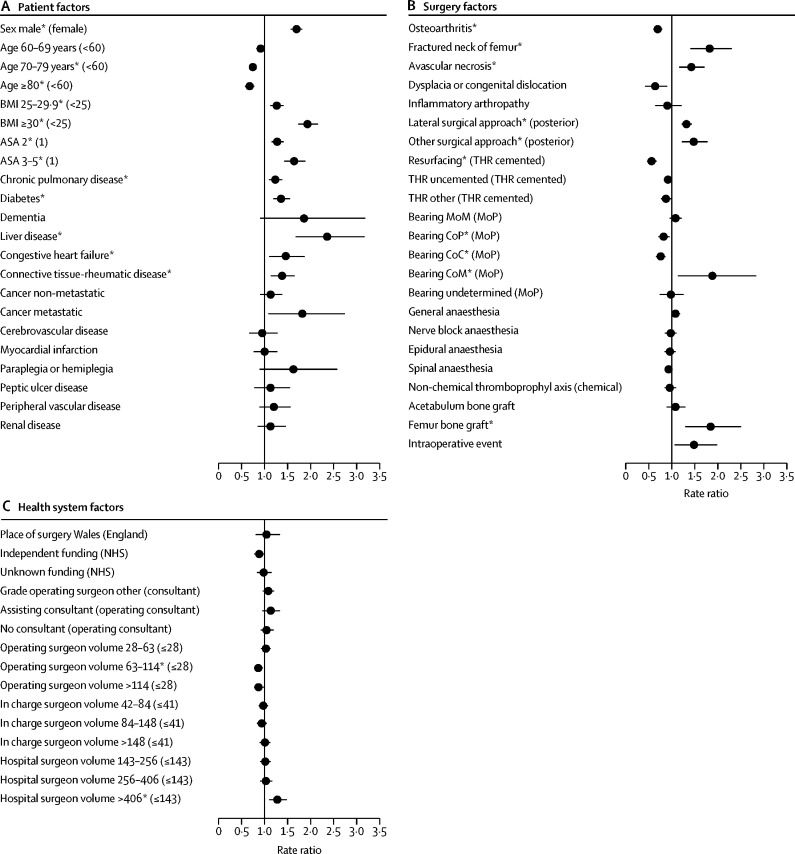
Risk factors of revision for prosthetic joint infection for the overall postoperative period, 2003–13 Reference category in parentheses. BMI=body-mass index. ASA=American Society of Anaesthesiologists. THR=total hip replacement. MoM=metal-on-metal. MoP=metal-on-polyethylene. CoP=ceramic-on-polyethylene. CoC=ceramic-on-ceramic. CoM=metal-on-ceramic. *Adjusted p value <0·05 (details in the appendix alongside the rate ratios and 95% CIs).

Men were at higher risk of revision for PJI in all time periods than women (figure 2). Over the entire follow-up, the risk was lower for patients older than 70 years than for patients younger than 60 years. However, this reduced risk was only observed after the first 6 months (appendix). BMI of 30 kg/m^2^ or higher was associated with an increased risk compared with BMI of less than 25 kg/m^2^. Patients with an ASA grade of 2 or higher were at greater risk than healthy patients (table). This was particularly evident during the first 6 months (appendix).

**Figure 3:**
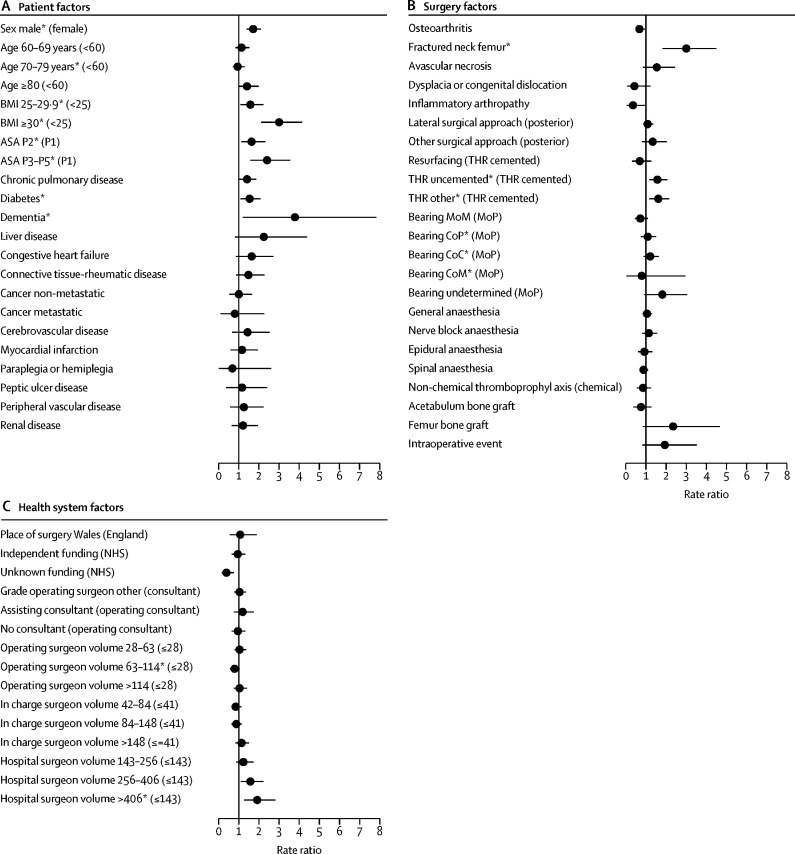
Risk factors of revision for prosthetic joint infection for the first 3 postoperative months Reference category in parentheses. BMI=body-mass index. ASA=American Society of Anaesthesiologists. THR=total hip replacement. MoM=metal-on-metal. MoP=metal-on-polyethylene. CoP=ceramic-on-polyethylene. CoC=ceramic-on-ceramic. CoM=metal-on-ceramic. *Adjusted p value <0·05 (details in the appendix alongside the rate ratios and 95% CIs).

Patients with a pre-existing history of chronic pulmonary disease, diabetes, liver disease, congestive heart failure, or connective tissue and rheumatologic diseases had a higher risk than did those without pre-existing history of these diseases (figure 2). Patients with diabetes or dementia were at increased risk of early revision for PJI (figure 3). Patients with liver disease were only at high risk beyond 24 months. No time-specific effect was observed for other comorbidities (appendix).

The risk varied according to the indication for the primary procedure. Patients operated on for osteoarthritis were less likely to be revised for PJI than those without osteoarthritis. Patients operated on for a fractured neck of femur, avascular necrosis (figure 2), or history of previous infection of the operated joint were at increased risk (table; appendix). A fractured neck of the femur was only associated with an increased risk of early revision for PJI (figure 3).

Operations done via a posterior surgical approach had the lowest risk of revision for PJI compared with other surgical approaches (figure 2). The surgical approach did not influence the early risk of revision for PJI (figure 3), but from 3 months onwards patients who had undergone a lateral approach were at higher risk (appendix).

Patients who had a primary hip resurfacing were at lower risk of revision for PJI (figure 2), but this lower risk was not evident in the first 3 postoperative months (figure 3). In the early postoperative period, patients who had undergone an uncemented, hybrid, or reverse hybrid total hip replacement (THR other, figure 3B) were at higher risk than those with cemented implants but from 3 to 24 months, they were at lower or similar risk (appendix). Further analysis showed a higher early risk of revision in patients with hybrid implant THRs (RR_<3mth_ 1·7, 95% CI 1·2–2·3) than in those with reverse hybrid implants (0·9, 0·4–2·0).

The risk of revision for PJI was also influenced by the type of bearing surfaces and this varied according to the time period. In the early postoperative period, no differences were observed (figure 3). Between 3 and 24 months, metal-on-metal THRs had a lower or similar risk than did metal-on-polyethylene THRs; beyond 24 months, the risk was higher for metal-on-metal (appendix). When the model was further adjusted for the type of surgery (resurfacing and THR cemented or not) the higher revision risk for PJI in the metal-on-metal group was identified earlier, from 12 months postoperation onwards (RR_12–24mth_ 1·8, 95% CI 1·3–2·3; RR_>24mth_ 2·2, 1·8–2·6). Ceramic-on-ceramic and ceramic-on-polyethylene surfaces were associated with a lower risk of long-term revision (from 12 months for ceramic-on-ceramic and 24 months for ceramic-on-polyethylene postoperation onwards) than metal-on-polyethylene bearings, which also had a higher risk of long-term revision for PJI (appendix).

Little or no difference in the risk of revision for PJI was found for the choice of anaesthetic technique, thromboprophylaxis regime, use of acetabular bone graft, or experience of intraoperative complication (Figure 2, Figure 3; appendix) Patients who received a femoral bone graft during the primary procedure were at higher risk of PJI with no evidence of a postoperative period-specific effect (figure 3; appendix).

The risk of revision for PJI was not different between Wales and England nor between the funding sources of the primary procedure (figure 2).

Revision for PJI was not influenced by the grade of the operating surgeon and the presence or absence of a consultant surgeon during surgery (figure 2).

Operating surgeons who had performed over 63 procedures in the 12 months preceding the primary surgery were weakly associated with a lower risk of revision for PJI than surgeons with a lower volume (figure 2). This pattern was inconsistent between time-periods and did not influence the early risk of revision for PJI (figure 3; appendix). The volume of all hip procedures done by the surgeon in charge of the surgery did not affect the risk of revision (figure 2). The risk of revision for PJI was higher in the first 3 months after primary surgery in hospitals that had performed over 255 hip procedures in the 12 months preceding the primary surgery than with hospitals with a small volume of activity (figure 3). No specific difference in the rate ratios were found beyond this period or for units with lower volumes of hip procedures (appendix).

## Discussion

At the patient level, men, younger patients, and those with high BMI or high ASA grades had an increased risk of revision for PJI. Comorbidities that increased the risk of revision for PJI included chronic pulmonary disease, diabetes, dementia, liver disease, congestive heart failure, and connective tissue or rheumatic diseases. These comorbidities and elevated BMI can potentially be optimised before surgery. A targeted preoperative intervention for male patients with high BMI and specific comorbidities seems particularly relevant.

At the surgical level, patients undergoing THR for fractured neck of femur or avascular necrosis were at higher risk of revision for PJI. Patients with a fracture are different to those who have conditions such as osteoarthritis, generally being older with a higher risk of mortality.^21^ Conditions that cause avascular necrosis, such as steroid use or irradiation, cause immunosuppression and also predispose towards PJI. The markedly higher risk in those with historical infection of the hip is novel, though unsurprising, and might be due to quiescent bacteria or other immune conditions that predispose to PJI. Lateral surgical approach and use of femoral bone graft also increased the risk. The increased risk with the lateral surgical approach is a novel finding that we postulate is due to increased tissue damage and bleeding caused by violating the abductor mechanism. Previous studies have suggested that the lateral approach is associated with more bleeding,^22^ worse patient related outcomes,^23^ and higher mortality.^24^ Approximately one third of hip replacements undertaken in England and Wales in 2016, still utilised this approach—although its use is declining.^21^ Early revision for PJI was higher in those receiving uncemented than cemented implants independent of bearing surface. At later time points, the risk was lower for uncemented THRs and resurfacings. This might reflect an initial protective effect of antibiotic impregnated bone cement. Long-term risk was higher in metal-on-metal bearings, possibly due to the soft tissue destruction associated with these implants,^25^ and was lower in bearings that included ceramic heads, which is concordant with a report^26^ from the Medicare population in the USA. In this Medicare population, ceramic bearings were used in younger and healthier patients. Our study adjusted for age and health status, which should mitigate the effects of any selection bias. A meta-analysis^27^ also showed weak evidence of reduced risk of PJI for ceramic bearing surfaces.

Factors at the health-care system level appear to be less important with no marked sustained associations across the time periods studied.

Consistent with previous studies,^10^, ^12^, ^28^ we observed higher risk in men and patients with high BMI. Contrary to previous findings,^10^, ^12^ younger patients were at higher risk, which could reflect the increased follow up in our study. Older patients could be at lower risk due to a propensity to non-operative management of PJI in this group. Smoking has previously been identified as a risk factor,^10^, ^29^ and although we did not have information on smoking habit, the surrogate comorbidity of chronic pulmonary disease was associated with increased risk. Evidence of an association between alcohol intake and increased risk has been inconsistent.^30^, ^31^ We observed higher risk in patients with liver disease, but this might represent several pathologies. Our study corroborates the previous findings^10^, ^30^, ^32^ of increased risk in patients with diabetes, rheumatoid arthritis, and congestive heart failure. We have shown for the first time that dementia is associated with an increased risk of early revision for PJI, which might reflect the high prevalence of other comorbidities in these patients.

The current study has several strengths. To our knowledge, this is the largest and most comprehensive investigation of several patient, surgical, and health-care related factors and their risk for revision for PJI of the hip. We used a large-scale cohort design comprising more participants (n=623 253) than those of the most up-to-date review on the topic (n=512 508 hip and knee replacements).^10^ Other strengths include the longer term follow-up of the cohort (median 4·6 years) and cutting-edge statistical analyses, which include the assessment of the effects of these potential risk factors at time-specific periods.

Our study has some limitations. Although pros-pectively collected, our data is observational and we can only draw inferences on the nature and magnitude of the associations but cannot establish causation. In the UK, no national gold standards have been agreed upon that are available to orthopaedic surgeons to diagnose PJI. As such, the reported indication of PJI in the NJR might vary between units but is reflective of contemporary practice with raised inflammatory markers, joint specific symptoms, sinuses, and positive microbiological cultures being used to diagnose PJI.^13^ The PJI diagnosis reflects a clinical judgment sufficient to lead the surgeon to conduct a very severe and invasive procedure tailored to tackle a PJI. Issues relating to under-reporting of revision for PJI, and thus potentially lower incidence estimates, are acknowledged.^33^ Linkage of the NJR data to microbiology data could reduce any misdiagnoses of PJI but has proven to be of limited generalisability with 12% NJR linkage achievable.^34^

The associations we have identified might vary with different causative pathogens, but unfortunately we do not have the data to explore this. Our findings should be considered as conservative estimates of the risk factors with the strongest effects. The investigations of the effect of comorbidities were limited to a subset of NJR patients linked to HES. This subset had higher ASA grades and therefore higher rate of revision for PJI than those excluded from these investigations, but they did not differ in terms of age, sex, BMI, or surgical characteristics, suggesting little evidence of differential selection bias. All other factors were investigated on the entire sample.

We have done appropriate modelling to adjust for known relevant confounders but residual confounding is still possible. We had no specific data on confounders, such as smoking and alcohol consumption, but have surrogate markers, such as chronic pulmonary disease and liver disease. BMI was not collected in the early years of the registry necessitating imputation of the data as with a previous study on this dataset.^24^ Competing risk due to revision for another cause or death, which in combination affected 55% of the primary hip replacements in the dataset during the period of observation, could not be accounted for in the modelling strategy. This was a pragmatic decision because we chose a strategy focusing on time-specific effects while accounting for the clustering nature of the data to disentangle the effect associated with surgical factors (likely to be more marked in the short-term to mid-term follow-up period) from those associated with health-risk behaviour (likely to be more marked in the mid-term to long-term follow-up period). This strategy was optimal because evidence supports non-proportional hazard rates. Finally, it was not possible to investigate any ethnic disparities in terms of revision for PJI due to the insufficient number of ethnic minority patients revised for PJI.

Preventive strategies for PJI largely focus on hygiene, use of protective equipment, management of care equipment and occupational exposure, and safe care of linen, the environment, and waste.^35^ Combinations of systemic antibiotics, antibiotic-impregnated cement, and conventional operating theatre ventilation are considered cost effective for preventing PJI.^35^ Identification of patient factors associated with increased need for revision for PJI can further guide the development of interventions and help target the provision of appropriate preventative care.

Using the largest longitudinal sample of primary hip replacements, we have shown several modifiable and non-modifiable factors to be associated with the risk of revision for a PJI after a primary hip replacement. For patients about to have hip replacement, identification of modifiable factors, use of targeted interventions, and beneficial modulation of some of these factors could be effective in reducing the incidence of PJI. It is important for clinicians to consider the non-modifiable factors, and the factors that exhibit time-specific effects on the risk of PJI, to counsel patients appropriately preoperatively.
