# Supplementary material for: Risk factors associated with revision for prosthetic joint infection after hip replacement: a prospective observational cohort study
Source: Lancet Infect Dis. 2018 Sep;18(9):1004–14. doi: 10.1016/S1473-3099(18)30345-1 (PMC6105575; doi:10.1016/S1473-3099(18)30345-1)
Supplement: Supplementary appendix [file mmc1.pdf]

THE LANCET Infectious Diseases

Supplementary webappendix

This webappendix formed part of the original submission and has been peer reviewed. We post it as supplied by the authors.

Supplement to: Lenguerrand E, Whitehouse MR, Beswick AD, et al. Risk factors associated with revision for prosthetic joint infection after hip replacement: a prospective observational cohort study. *Lancet Infect Dis* 2018; published online July 25. http://dx.doi.org/10.1016/S1473-3099(18)30345-1.

Web extra materials

Appendix 1

Patients with incompletely registered two-stage procedures did not differ from those with complete information at the time of their index surgery in terms of age (≥80y: 6% vs 6%; ≤60y: 30% vs 29%), sex (female: 43% vs 46%), BMI (<25kg/m^2: 16% vs 13%;≥30kg/m^2: 55% vs 55%), American Society of Anaesthesiologists (ASA) Physical Status grade (>P2: 16% vs 15%), or type of surgery (uncemented total hip replacement: 39% vs 42%; resurfacing: 9% vs 7%).

For patients with incompletely registered two-stage revision procedures, the date of the first revision operation and the period they had been at risk of revision for PJI were estimated. We initially derived the relative weight of time elapsed between the index and the first operation by year and type of index surgery using patients with complete information: 100 x (length of $\text{time}_{\text{index surgery- 1st operation}}$/length of $\text{time}_{\text{index surgery- 2nd operation}}$). We then applied these weights to the length of time between the index and the second procedure for those with incomplete information to obtain an estimated "index surgery–1st operation" duration.

Appendix 2

Data on comorbidity were derived from the HES records. HES hospital admissions were linked to the NJR using a common anonymised patient identifier provided to NJR end-user by Northgate. The presence of each of the following comorbidities was identified using the ICD-10 codes associated with each hospital admission which occurred in the five years preceding the date of the primary replacement recorded in the NJR. The PEDW records were similarly linked but the dates of hospital admission were either missing or incomplete in the data extract available to us for numerous records preventing the accurate derivation of the five-year comorbidity presence. The comorbidity profile of patients with records in PEDW and/or operated in Wales could not be established with accuracy.

The analysis of the effect of specific comorbidity on revision for PJI was therefore restricted to patients with a hip replacement performed in England with linked HES record(s), with no record in the PEDW and no evidence of residency outside England. In other words, patients with their hip replacement performed in England but with PEDW record(s), patients with their hip replacement performed in Wales with or without PEDW/HES record(s) and patients with evidence of residency outside England were excluded. Moreover to reduce a risk of misclassification bias, patients operated in England with unlinked HES or PEDW record(s) could not be included in these analyses, i.e. not accounted as healthy patients: It was impossible to disentangle those who were genuinely healthy (i.e. absence of HES and PEDW record=true absence of any hospitalisation) from those who had been admitted to the hospital for any reason but for whom linkage between their NJR and HES/PEDW records could not be established(either due to missing data in the HES/PEDW file or of absence of match with the linkage method used by Northgate). No major evidence of difference was identified between included (n=495,456, Figure1) and excluded (n=623,253-495,456=127,797, figure1) patients: median age 68y vs 69y, Male=42% vs 40%, median BMI 27 vs 28. However, those included had worse ASA grade (healthy patient(ASA=1): 17% vs 25%; mild systemic disease (ASA=2): 68% vs 4%; severe systemic disease (ASA=3): 15% vs 10%). This is expected as patients with HES records have at least one comorbidity and as explained above, some of the patients without a HES record are "healthy" patients with no comorbidity requiring hospital inpatient admission or hospital outpatient visit."

The comorbidities considered were derived from the Charlson comorbidity index using the following ICD-10 codes:[15]

- Myocardial infarction: I21.x, I22.x, I25.2
- Congestive heart failure: I09.9, I11.0, I13.0, I13.2, I25.5, I42.0, I42.5–I42.9, I43.x, I50.x, P29.0
- Peripheral vascular disease: I70.x, I71.x, I73.1, I73.8, I73.9, I77.1, I79.0, I79.2, K55.1, K55.8, K55.9, Z95.8, Z95.9
- Cerebrovascular disease: G45.x, G46.x, H34.0, I60.x–I69.x
- Dementia: F00.x–F03.x, F05.1, G30.x, G31.1
- Chronic pulmonary disease: I27.8, I27.9, J40.x–J47.x, J60.x–J67.x, J68.4, J70.1, J70.3
- Connective tissue disease or rheumatic disease: M05.x, M06.x, M31.5, M32.x–M34.x, M35.1, M35.3, M36.0
- Peptic ulcer disease: K25.x–K28.x
- Liver disease(mild and moderate or severe): B18.x, K70.0–K70.3, K70.9, K71.3–K71.5, K71.7, K73.x, K74.x, K76.0, K76.2–K76.4, K76.8, K76.9, Z94.4 + I85.0, I85.9, I86.4, I98.2, K70.4, K71.1, K72.1, K72.9, K76.5, K76.6, K76.7
- Diabetes (with and without chronic complication): E10.0, E10.1, E10.6, E10.8, E10.9, E11.0, E11.1, E11.6, E11.8, E11.9, E12.0, E12.1, E12.6, E12.8, E12.9, E13.0, E13.1, E13.6, E13.8, E13.9, E14.0, E14.1, E14.6, E14.8, E14.9+ E10.2–E10.5, E10.7, E11.2–E11.5, E11.7, E12.2–E12.5, E12.7, E13.2–E13.5, E13.7, E14.2–E14.5, E14.7
- Hemiplegia or paraplegia: G04.1, G11.4, G80.1, G80.2, G81.x, G82.x, G83.0–G83.4, G83.9
- Renal disease: I12.0, I13.1, N03.2–N03.7, N05.2– N05.7, N18.x, N19.x, N25.0, Z49.0– Z49.2, Z94.0, Z99.2
- Cancer(any malignancy and metastatic solid tumor): C00.x–C26.x, C30.x–C34.x, C37.x– C41.x, C43.x, C45.x–C58.x, C60.x– C76.x, C81.x–C85.x, C88.x, C90.x–C97.x+ C77.x–C80.x
- AIDS/HIV: B20.x–B22.x, B24.x

HIV/AIDS as a comorbidity group was not considered further in this research as only 45 patients had been admitted for this indication with none revised for a PJI

Appendix 3

Appendix Table 1: Sample description and incidence rates by period of revision for PJI from the index procedures

		0–3 months				3–6 months			
		Person-years	Cases	Incidence	95%CI	Person-years	Cases	Incidence	95%CI
**Sex**	**Female**	91,233	173	1·90	[1·62 to 2·20]	90,739	74	0·82	[0·64 to 1·02]
	**Male**	61,453	199	3·24	[2·80 to 3·72]	61,033	130	2·13	[1·78 to 2·53]
**Age**	**<60**	32,351	73	2·26	[1·77 to 2·84]	32,215	42	1·30	[0·94 to 1·76]
	**[60–69]**	46,898	123	2·62	[2·18 to 3·13]	46,697	65	1·39	[1·07 to 1·77]
	**[70–79]**	51,548	112	2·17	[1·79 to 2·61]	51,253	82	1·60	[1·27 to 1·99]
	**≥80**	21,889	64	2·92	[2·25 to 3·73]	21,608	15	0·69	[0·39 to 1·14]
**Ethnicity**	**White**	114,906	310	2·70	[2·41 to 3·02]	114,184	166	1·45	[1·24 to 1·69]
	**Black Afr. origin**	698	0			695	1	1·44	[0·04 to 8·01]
	**South Asian**	392	1	2·55	[0·06 to 14·20]	389	1	2·57	[0·07 to 14·34]
	**Other and mixed**	793	0			787	4	5·08	[1·38 to 13·01]
	**Unclear**	4,559	5	1·10	[0·36 to 2·56]	4,534	2	0·44	[0·05 to 1·59]
**BMI[1]**	**<25**	17,537	26	1·48	[0·97 to 2·17]	17,427	12	0·69	[0·36 to 1·20]
	**[25–29·9]**	32,640	72	2·21	[1·73 to 2·78]	32,488	44	1·35	[0·98 to 1·82]
	**≥30**	30,848	140	4·54	[3·82 to 5·36]	30,689	71	2·31	[1·81 to 2·92]
	**Missing**	71,659	134	1·87	[1·57 to 2·21]	71,168	77	1·08	[0·85 to 1·35]
**ASA[2]**	**P1**	28,081	38	1·35	[0·96 to 1·86]	27,993	19	0·68	[0·41 to 1·06]
	**P2**	102,584	245	2·39	[2·10 to 2·71]	102,087	151	1·48	[1·25 to 1·73]
	**P3–P5**	22,020	89	4·04	[3·25 to 4·97]	21,692	34	1·57	[1·09 to 2·19]
**Chronic Pulmonary Disease**	**No**	106,087	262	2·47	[2·18 to 2·79]	105,463	152	1·44	[1·22 to 1·69]
	**Yes**	15,260	54	3·54	[2·66 to 4·62]	15,126	22	1·45	[0·91 to 2·20]
**Diabetes**	**No**	110,996	271	2·44	[2·16 to 2·75]	110,338	148	1·34	[1·13 to 1·58]
	**Yes**	10,351	45	4·35	[3·17 to 5·82]	10,251	26	2·54	[1·66 to 3·72]
**Dementia**	**No**	120,850	311	2·57	[2·30 to 2·88]	120,108	173	1·44	[1·23 to 1·67]
	**Yes**	497	5	10·06	[3·27 to 23·47]	481	1	2·08	[0·05 to 11·59]
**Liver Disease**	**No**	120,373	310	2·58	[2·30 to 2·88]	119,634	172	1·44	[1·23 to 1·67]
	**Yes**	974	6	6·16	[2·26 to 13·40]	955	2	2·09	[0·25 to 7·56]
**Congestive Heart Failure**	**No**	118,777	303	2·55	[2·27 to 2·86]	118,081	174	1·47	[1·26 to 1·71]
	**Yes**	2,570	13	5·06	[2·69 to 8·65]	2,508	0		
**Connective Tissue-Rheumatologic Disease**	**No**	116,004	297	2·56	[2·28 to 2·87]	115,299	160	1·39	[1·18 to 1·62]
	**Yes**	5,343	19	3·56	[2·14 to 5·55]	5,290	14	2·65	[1·45 to 4·44]
**Cancer**	**No**	115,909	301	2·6	[2·31 to 2·91]	115,283	164	1·42	[1·21 to 1·66]
	**Non-metastatic**	4,526	13	2·87	[1·53 to 4·91]	4,477	8	1·79	[0·77 to 3·52]
	**Metastatic**	912	2	2·19	[0·27 to 7·92]	830	2	2·41	[0·29 to 8·71]
**Cerebrovascular Disease**	**No**	118,939	306	2·57	[2·29 to 2·88]	118,222	171	1·45	[1·24 to 1·68]
	**Yes**	2,409	10	4·15	[1·99 to 7·64]	2,367	3	1·27	[0·26 to 3·70]
**Myocardial Infarction**	**No**	118,083	304	2·57	[2·29 to 2·88]	117,374	171	1·46	[1·25 to 1·69]
	**Yes**	3,264	12	3·68	[1·90 to 6·42]	3,215	3	0·93	[0·19 to 2·73]
**Paraplegia and Hemiplegia**	**No**	120,854	315	2·61	[2·33 to 2·91]	120,106	172	1·43	[1·23 to 1·66]
	**Yes**	493	1	2·03	[0·05 to 11·30]	483	2	4·14	[0·50 to 14·97]
**Peptic Ulcer Disease**	**No**	119,775	311	2·6	[2·32 to 2·90]	119,037	172	1·44	[1·24 to 1·68]
	**Yes**	1,572	5	3·18	[1·03 to 7·42]	1,552	2	1·29	[0·16 to 4·65]
**Peripheral Vascular Disease**	**No**	118,989	307	2·58	[2·30 to 2·89]	118,268	170	1·44	[1·23 to 1·67]
	**Yes**	2,358	9	3·82	[1·75 to 7·24]	2,321	4	1·72	[0·47 to 4·41]
**Renal Disease**	**No**	117,515	302	2·57	[2·29 to 2·88]	116,827	165	1·41	[1·21 to 1·65]
	**Yes**	3,832	14	3·65	[2·00 to 6·13]	3,762	9	2·39	[1·09 to 4·54]
**Osteoarthritis**	**No**	10,612	40	3·77	[2·69 to 5·13]	10,425	25	2·40	[1·55 to 3·54]
	**Yes**	142,074	332	2·34	[2·09 to 2·60]	141,348	179	1·27	[1·09 to 1·47]
**Fractured neck of femur**	**No**	149,655	350	2·34	[2·10 to 2·60]	148,821	197	1·32	[1·15 to 1·52]
	**Yes**	3,030	22	7·26	[4·55 to 10·99]	2,952	7	2·37	[0·95 to 4·89]
**Previous hip infection**	**No**	152,526	369	2·42	[2·18 to 2·68]	151,616	199	1·31	[1·14 to 1·51]

	**Yes**	159	3	18·83	[3·88 to 55·02]	156	5	32·03	[10·40 to 74·74]
**Avascular necrosis**	**No**	148,793	357	2·4	[2·16 to 2·66]	147,920	198	1·34	[1·16 to 1·54]
	**Yes**	3,893	15	3·85	[2·16 to 6·36]	3,853	6	1·56	[0·57 to 3·39]
**Dysplasia/Congenital dislocation**	**No**	150,348	370	2·46	[2·22 to 2·72]	149,445	202	1·35	[1·17 to 1·55]
	**Yes**	2,337	2	0·86	[0·10 to 3·09]	2,327	2	0·86	[0·10 to 3·10]
**Inflammatory arthropathy**	**No**	150,445	370	2·46	[2·22 to 2·72]	149,547	199	1·33	[1·15 to 1·53]
	**Yes**	2,240	2	0·89	[0·11 to 3·22]	2,225	5	2·25	[0·73 to 5·24]
**Surgical approach**	**Posterior**	82,622	201	2·43	[2·11 to 2·79]	82,147	91	1·11	[0·89 to 1·36]
	**Lateral**	63,060	150	2·38	[2·01 to 2·79]	62,659	104	1·66	[1·36 to 2·01]
	**Other**	7,004	21	3·00	[1·86 to 4·58]	6,966	9	1·29	[0·59 to 2·45]
**Procedure[3]**	**Resurfacing**	8,955	10	1·12	[0·54 to 2·05]	8,909	6	0·67	[0·25 to 1·47]
	**THR cemented**	56,101	101	1·80	[1·47 to 2·19]	55,708	91	1·63	[1·32 to 2·01]
	**THR uncemented**	59,096	171	2·89	[2·48 to 3·36]	58,789	77	1·31	[1·03 to 1·64]
	**THR other**	28,533	90	3·15	[2·54 to 3·88]	28,367	30	1·06	[0·71 to 1·51]
**Type of bearing[4]**	**MoP**	89,893	217	2·41	[2·10 to 2·76]	89,261	133	1·49	[1·25 to 1·77]
	**MoM**	16,859	28	1·66	[1·10 to 2·40]	16,776	13	0·77	[0·41 to 1·33]
	**CoP**	18,065	43	2·38	[1·72 to 3·21]	17,991	18	1.00	[0·59 to 1·58]
	**CoC**	24,447	71	2·90	[2·27 to 3·66]	24,353	34	1·40	[0·97 to 1·95]
	**CoM**	556	1	1·80	[0·05 to 10·02]	554	1	1·81	[0·05 to 10·06]
	**Other**	2,865	12	4·19	[2·16 to 7·32]	2,838	5	1·76	[0·57 to 4·11]
**General Anaesthesia**	**No**	79,324	193	2·43	[2·10 to 2·80]	78,875	96	1·22	[0·99 to 1·49]
	**Yes**	73,361	179	2·44	[2·10 to 2·82]	72,897	108	1·48	[1·22 to 1·79]
**Nerve Block Anaesthesia**	**No**	136,949	328	2·40	[2·14 to 2·67]	136,143	180	1·32	[1·14 to 1·53]
	**Yes**	15,736	44	2·80	[2·03 to 3·75]	15,630	24	1·54	[0·98 to 2·28]
**Epidural Anaesthesia**	**No**	139,266	341	2·45	[2·20 to 2·72]	138,437	183	1·32	[1·14 to 1·53]
	**Yes**	13,419	31	2·31	[1·57 to 3·28]	13,335	21	1·57	[0·97 to 2·41]
**Spinal Anaesthesia**	**No**	59,908	154	2·57	[2·18 to 3·01]	59,512	93	1·56	[1·26 to 1·91]
	**Yes**	92,778	218	2·35	[2·05 to 2·68]	92,261	111	1·20	[0·99 to 1·45]
**Thromboprophylaxis regimen**	**Chemical**	137,898	343	2·49	[2·23 to 2·77]	137,074	182	1·33	[1·14 to 1·54]
	**Non chemical**	14,787	29	1·96	[1·31 to 2·82]	14,698	22	1·50	[0·94 to 2·27]
**Acetabulum bonegraft**	**No**	146,384	361	2·47	[2·22 to 2·73]	145,515	193	1·33	[1·15 to 1·53]
	**Yes**	6,302	11	1·75	[0·87 to 3·12]	6,257	11	1·76	[0·88 to 3·15]
**Femur bonegraft**	**No**	151,500	366	2·42	[2·17 to 2·68]	150,596	203	1·35	[1·17 to 1·55]
	**Yes**	1,185	6	5·06	[1·86 to 11·02]	1,177	1	0·85	[0·02 to 4·74]
**Intra operative event**	**No**	150,895	364	2·41	[2·17 to 2·67]	150,003	201	1·34	[1·16 to 1·54]
	**Yes**	1,790	8	4·47	[1·93 to 8·80]	1,770	3	1·70	[0·35 to 4·95]
**Place of surgery**	**England**	144,076	349	2·42	[2·17 to 2·69]	143,221	193	1·35	[1·16 to 1·55]
	**Wales**	8,609	23	2·67	[1·69 to 4·01]	8,551	11	1·29	[0·64 to 2·30]
**Funding**	**NHS**	124,108	322	2·59	[2·32 to 2·89]	123,340	166	1·35	[1·15 to 1·57]
	**Independent**	22,236	43	1·93	[1·40 to 2·60]	22,131	29	1·31	[0·88 to 1·88]
	**Unspecified**	6,341	7	1·10	[0·44 to 2·27]	6,302	9	1·43	[0·65 to 2·71]
**Grade operating surgeon**	**Consultant**	129,057	309	2·39	[2·13 to 2·68]	128,296	170	1·33	[1·13 to 1·54]
	**Other**	23,629	63	2·67	[2·05 to 3·41]	23,476	34	1·45	[1·00 to 2·02]
**Consultant involvement**	**Operating**	129,057	309	2·39	[2·13 to 2·68]	128,296	170	1·33	[1·13 to 1·54]
	**Assisting**	8,145	26	3·19	[2·09 to 4·68]	8,090	15	1·85	[1·04 to 3·06]
	**Not involved**	15,484	37	2·39	[1·68 to 3·29]	15,387	19	1·23	[0·74 to 1·93]
**Total volume Operating surgeon**	**≤28**	40,270	105	2·61	[2·13 to 3·16]	40,004	63	1·57	[1·21 to 2·01]
	**]28–63]**	38,787	103	2·66	[2·17 to 3·22]	38,540	59	1·53	[1·17 to 1·97]
	**]63–114]**	37,685	74	1·96	[1·54 to 2·47]	37,474	46	1·23	[0·90 to 1·64]
	**>114**	35,944	90	2·50	[2·01 to 3·08]	35,754	36	1·01	[0·71 to 1·39]
**Total volume Surgeon in charge**	**≤41**	40,610	105	2·59	[2·11 to 3·13]	40,341	64	1·59	[1·22 to 2·03]
	**]41–84]**	38,735	85	2·19	[1·75 to 2·71]	38,494	58	1·51	[1·14 to 1·95]
	**]84–148]**	37,294	84	2·25	[1·80 to 2·79]	37,086	43	1·16	[0·84 to 1·56]
	**>148**	36,046	98	2·72	[2·21 to 3·31]	35,851	39	1·09	[0·77 to 1·49]

**Total volume Hospital**	**≤143**	39,288	71	1·81	[1·41 to 2·28]	39,054	53	1·36	[1·02 to 1·78]
	**]143–256]**	38,723	81	2·09	[1·66 to 2·60]	38,505	41	1·06	[0·76 to 1·44]
	**]256–406]**	37,857	102	2·69	[2·20 to 3·27]	37,607	49	1·30	[0·96 to 1·72]
	**>406**	36,817	118	3·21	[2·65 to 3·84]	36,607	61	1·67	[1·27 to 2·14]

(Continues on next page)

		6–12 months				12–24 months				≥24mths			
		**Person-years**	**Cases**	**Incidence**	**95%CI**	**Person-years**	**Cases**	**Incidence**	**95%CI**	**Person-years**	**Cases**	**Incidence**	**95%CI**
**Sex**	**Female**	185,292	158	0·85	[0·72 to 1·00]	338,416	276	0·82	[0·72 to 0·92]	1,144,012	562	0·49	[0·45 to 0·53]
	**Male**	124,427	216	1·74	[1·51 to 1·98]	226,723	336	1·48	[1·33 to 1·65]	763,534	581	0·76	[0·70 to 0·83]
**Age**	**<60**	65,889	94	1·43	[1·15 to 1·75]	121,143	163	1·35	[1·15 to 1·57]	436,403	367	0·84	[0·76 to 0·93]
	**[60–69]**	95,482	154	1·61	[1·37 to 1·89]	175,086	217	1·24	[1·08 to 1·42]	613,800	383	0·62	[0·56 to 0·69]
	**[70–79]**	104,572	101	0·97	[0·79 to 1·17]	190,787	170	0·89	[0·76 to 1·04]	635,691	317	0·50	[0·45 to 0·56]
	**≥80**	43,777	25	0·57	[0·37 to 0·84]	78,123	62	0·79	[0·61 to 1·02]	221,653	76	0·34	[0·27 to 0·43]
**Ethnicity**	**White**	232,895	317	1·36	[1·22 to 1·52]	423,018	522	1·23	[1·13 to 1·34]	1,371,672	993	0·72	[0·68 to 0·77]
	**Black Afr. origin**	1,421	1	0·70	[0·02 to 3·92]	2,575	2	0·78	[0·09 to 2·81]	7,762	8	1·03	[0·44 to 2·03]
	**South Asian**	794	0			1,410	2	1·42	[0·17 to 5·12]	4,238	2	0·47	[0·06 to 1·70]
	**Other and mixed**	1,609	4	2·49	[0·68 to 6·37]	2,893	1	0·35	[0·01 to 1·93]	8,324	5	0·60	[0·20 to 1·40]
	**Unclear**	9,276	3	0·32	[0·07 to 0·95]	16,836	5	0·30	[0·10 to 0·69]	61,229	10	0·16	[0·08 to 0·30]
**BMI**[1]	**<25**	35,544	46	1·29	[0·95 to 1·73]	63,117	59	0·93	[0·71 to 1·21]	164,061	129	0·79	[0·66 to 0·93]
	**[25–29·9]**	66,375	65	0·98	[0·76 to 1·25]	118,125	127	1·08	[0·90 to 1·28]	308,197	272	0·88	[0·78 to 0·99]
	**≥30**	62,672	139	2·22	[1·86 to 2·62]	111,221	217	1·95	[1·70 to 2·23]	281,847	374	1·33	[1·20 to 1·47]
	**missing**	145,128	124	0·85	[0·71 to 1·02]	272,675	209	0·77	[0·67 to 0·88]	1,153,442	368	0·32	[0·29 to 0·35]
**ASA**[2]	**P1**	57,323	52	0·91	[0·68 to 1·19]	106,866	99	0·93	[0·75 to 1·13]	436,795	274	0·63	[0·56 to 0·71]
	**P2**	208,551	244	1·17	[1·03 to 1·33]	380,399	404	1·06	[0·96 to 1·17]	1,242,401	728	0·59	[0·54 to 0·63]
	**P3–P5**	43,845	78	1·78	[1·41 to 2·22]	77,873	109	1·40	[1·15 to 1·69]	228,351	141	0·62	[0·52 to 0·73]
**Chronic Pulmonary Disease**	**No**	215,232	276	1·28	[1·14 to 1·44]	392,341	451	1·15	[1·05 to 1·26]	1,308,147	923	0·71	[0·66 to 0·75]
	**Yes**	30,763	49	1·59	[1·18 to 2·11]	54,391	81	1·49	[1·18 to 1·85]	145,078	95	0·65	[0·53 to 0·80]
**Diabetes**	**No**	225,168	282	1·25	[1·11 to 1·41]	409,791	471	1·15	[1·05 to 1·26]	1,353,215	948	0·70	[0·66 to 0·75]
	**Yes**	20,827	43	2·06	[1·49 to 2·78]	36,942	61	1·65	[1·26 to 2·12]	100,010	70	0·70	[0·55 to 0·88]
**Dementia**	**No**	245,039	323	1·32	[1·18 to 1·47]	445,124	531	1·19	[1·09 to 1·30]	1,450,077	1,017	0·70	[0·66 to 0·75]
	**Yes**	956	2	2·09	[0·25 to 7·56]	1,608	1	0·62	[0·02 to 3·46]	3,148	1	0·32	[0·01 to 1·77]
**Liver Disease**	**No**	244,073	318	1·30	[1·16 to 1·45]	443,475	524	1·18	[1·08 to 1·29]	1,445,328	1,003	0·69	[0·65 to 0·74]
	**Yes**	1,922	7	3·64	[1·46 to 7·50]	3,257	8	2·46	[1·06 to 4·84]	7,897	15	1·90	[1·06 to 3·13]
**Congestive Heart Failure**	**No**	240,968	311	1·29	[1·15 to 1·44]	438,055	519	1·18	[1·09 to 1·29]	1,431,079	1,000	0·70	[0·66 to 0·74]
	**Yes**	5,027	14	2·79	[1·52 to 4·67]	8,678	13	1·50	[0·80 to 2·56]	22,146	18	0·81	[0·48 to 1·28]
**Connective Tissue-Rheumatologic Disease**	**No**	235,253	306	1·30	[1·16 to 1·45]	427,626	509	1·19	[1·09 to 1·30]	1,398,551	979	0·70	[0·66 to 0·75]
	**Yes**	10,742	19	1·77	[1·06 to 2·76]	19,106	23	1·20	[0·76 to 1·81]	54,674	39	0·71	[0·51 to 0·98]
**Cancer**	**No**	235,410	310	1·32	[1·17 to 1·47]	428,444	510	1·19	[1·09 to 1·30]	1,404,125	977	0·70	[0·65 to 0·74]
	**Non-metastatic**	9,047	11	1·22	[0·61 to 2·18]	15,873	18	1·13	[0·67 to 1·79]	43,765	35	0·80	[0·56 to 1·11]
	**Metastatic**	1,538	4	2·60	[0·71 to 6·66]	2,415	4	1·66	[0·45 to 4·24]	5,335	6	1·12	[0·41 to 2·45]
**Cerebrovascular Disease**	**No**	241,218	318	1·32	[1·18 to 1·47]	438,379	526	1·20	[1·10 to 1·31]	1,431,462	1,008	0·70	[0·66 to 0·75]
	**Yes**	4,777	7	1·47	[0·59 to 3·02]	8,353	6	0·72	[0·26 to 1·56]	21,763	10	0·46	[0·22 to 0·85]
**Myocardial Infarction**	**No**	239,489	319	1·33	[1·19 to 1·49]	435,606	516	1·18	[1·08 to 1·29]	1,420,342	995	0·70	[0·66 to 0·75]
	**Yes**	6,506	6	0·92	[0·34 to 2·01]	11,126	16	1·44	[0·82 to 2·34]	32,883	23	0·70	[0·44 to 1·05]
**Paraplegia and Hemiplegia**	**No**	245,018	323	1·32	[1·18 to 1·47]	445,000	527	1·18	[1·09 to 1·29]	1,448,438	1,014	0·70	[0·66 to 0·74]
	**Yes**	977	2	2·05	[0·25 to 7·40]	1,733	5	2·89	[0·94 to 6·73]	4,787	4	0·84	[0·23 to 2·14]
**Peptic Ulcer Disease**	**No**	242,858	319	1·31	[1·17 to 1·47]	441,133	527	1·19	[1·09 to 1·30]	1,435,838	1,004	0·70	[0·66 to 0·74]
	**Yes**	3,137	6	1·91	[0·70 to 4·16]	5,599	5	0·89	[0·29 to 2·08]	17,386	14	0·81	[0·44 to 1·35]
**Peripheral Vascular Disease**	**No**	241,311	314	1·30	[1·16 to 1·45]	438,560	523	1·19	[1·09 to 1·30]	1,432,496	1,004	0·70	[0·66 to 0·75]
	**Yes**	4,684	11	2·35	[1·17 to 4·20]	8,173	9	1·10	[0·50 to 2·09]	20,729	14	0·68	[0·37 to 1·13]
**Renal Disease**	**No**	238,411	317	1·33	[1·19 to 1·48]	434,249	523	1·20	[1·10 to 1·31]	1,430,542	1,004	0·70	[0·66 to 0·75]
	**Yes**	7,584	8	1·05	[0·46 to 2·08]	12,483	9	0·72	[0·33 to 1·37]	22,682	14	0·62	[0·34 to 1·04]
**Osteoarthritis**	**No**	21,046	45	2·14	[1·56 to 2·86]	37,179	53	1·43	[1·07 to 1·86]	110,018	86	0·78	[0·63 to 0·97]
	**Yes**	288,672	329	1·14	[1·02 to 1·27]	527,960	559	1·06	[0·97 to 1·15]	1,797,528	1,057	0·59	[0·55 to 0·62]
**Fractured neck of femur**	**No**	303,797	365	1·20	[1·08 to 1·33]	555,284	599	1·08	[0·99 to 1·17]	1,885,926	1,128	0·60	[0·56 to 0·63]
	**Yes**	5,921	9	1·52	[0·70 to 2·89]	9,854	13	1·32	[0·70 to 2·26]	21,620	15	0·69	[0·39 to 1·14]
**Previous hip infection**	**No**	309,402	371	1·20	[1·08 to 1·33]	564,575	607	1·08	[0·99 to 1·16]	1,905,687	1,137	0·60	[0·56 to 0·63]
	**Yes**	317	3	9·47	[1·95 to 27·68]	564	5	8·87	[2·88 to 20·71]	1,859	6	3·23	[1·18 to 7·02]
**Avascular necrosis**	**No**	301,902	356	1·18	[1·06 to 1·31]	550,948	588	1·07	[0·98 to 1·16]	1,857,651	1,098	0·59	[0·56 to 0·63]

	**Yes**	7,816	18	2·30	[1·36 to 3·64]	14,191	24	1·69	[1·08 to 2·52]	49,895	45	0·90	[0·66 to 1·21]
**Dysplasia/Congenital dislocation**	**No**	304,951	370	1·21	[1·09 to 1·34]	556,398	607	1·09	[1·01 to 1·18]	1,876,893	1,128	0·60	[0·57 to 0·64]
	**Yes**	4,768	4	0·84	[0·23 to 2·15]	8,740	5	0·57	[0·19 to 1·34]	30,653	15	0·49	[0·27 to 0·81]
**Inflammatory arthropathy**	**No**	305,185	365	1·20	[1·08 to 1·33]	556,822	604	1·08	[1·00 to 1·17]	1,878,372	1,127	0·60	[0·57 to 0·64]
	**Yes**	4,534	9	1·99	[0·91 to 3·77]	8,316	8	0·96	[0·42 to 1·90]	29,174	16	0·55	[0·31 to 0·89]
**Surgical approach**	**Posterior**	167,692	172	1·03	[0·88 to 1·19]	302,355	274	0·91	[0·80 to 1·02]	931,097	504	0·54	[0·50 to 0·59]
	**Lateral**	127,809	183	1·43	[1·23 to 1·65]	236,951	309	1·30	[1·16 to 1·46]	908,808	588	0·65	[0·60 to 0·70]
	**Other**	14,217	19	1·34	[0·80 to 2·09]	25,833	29	1·12	[0·75 to 1·61]	67,641	51	0·75	[0·56 to 0·99]
**Procedure[3]**	**Resurfacing**	18,236	15	0·82	[0·46 to 1·36]	35,345	34	0·96	[0·67 to 1·34]	173,640	109	0·63	[0·52 to 0·76]
	**THR cement**	113,522	144	1·27	[1·07 to 1·49]	207,937	241	1·16	[1·02 to 1·31]	763,434	437	0·57	[0·52 to 0·63]
	**THR uncemented**	120,099	137	1·14	[0·96 to 1·35]	218,190	256	1·17	[1·03 to 1·33]	648,021	433	0·67	[0·61 to 0·73]
	**THR other**	57,861	78	1·35	[1·07 to 1·68]	103,666	81	0·78	[0·62 to 0·97]	322,451	164	0·51	[0·43 to 0·59]
**Type of bearing[4]**	**MoP**	181,892	211	1·16	[1·01 to 1·33]	330,558	342	1·03	[0·93 to 1·15]	1,114,239	602	0·54	[0·50 to 0·59]
	**MoM**	34,308	43	1·25	[0·91 to 1·69]	66,609	110	1·65	[1·36 to 1·99]	313,058	332	1·06	[0·95 to 1·18]
	**CoP**	36,785	54	1·47	[1·10 to 1·92]	64,458	55	0·85	[0·64 to 1·11]	190,884	82	0·43	[0·34 to 0·53]
	**CoC**	49,835	53	1·06	[0·80 to 1·39]	90,454	90	0·99	[0·80 to 1·22]	239,512	94	0·39	[0·32 to 0·48]
	**CoM**	1,132	3	2·65	[0·55 to 7·74]	2,182	4	1·83	[0·50 to 4·69]	6,128	11	1·80	[0·90 to 3·21]
	**Other**	5,767	10	1·73	[0·83 to 3·19]	10,878	11	1·01	[0·50 to 1·81]	43,724	22	0·50	[0·32 to 0·76]
**General Anaesthesia**	**No**	160,978	180	1·12	[0·96 to 1·29]	290,668	320	1·10	[0·98 to 1·23]	922,355	528	0·57	[0·52 to 0·62]
	**Yes**	148,741	194	1·30	[1·13 to 1·50]	274,471	292	1·06	[0·95 to 1·19]	985,191	615	0·62	[0·58 to 0·68]
**Nerve Block Anaesthesia**	**No**	277,831	342	1·23	[1·10 to 1·37]	505,436	549	1·09	[1·00 to 1·18]	1,695,232	1,027	0·61	[0·57 to 0·64]
	**Yes**	31,887	32	1·00	[0·69 to 1·42]	59,702	63	1·06	[0·81 to 1·35]	212,314	116	0·55	[0·45 to 0·66]
**Epidural Anaesthesia**	**No**	282,528	331	1·17	[1·05 to 1·30]	513,753	552	1·07	[0·99 to 1·17]	1,678,953	1,008	0·60	[0·56 to 0·64]
	**Yes**	27,190	43	1·58	[1·14 to 2·13]	51,385	60	1·17	[0·89 to 1·50]	228,593	135	0·59	[0·50 to 0·70]
**Spinal Anaesthesia**	**No**	121,407	153	1·26	[1·07 to 1·48]	224,878	251	1·12	[0·98 to 1·26]	837,208	529	0·63	[0·58 to 0·69]
	**Yes**	188,312	221	1·17	[1·02 to 1·34]	340,260	361	1·06	[0·95 to 1·18]	1,070,339	614	0·57	[0·53 to 0·62]
**Thromboprophylaxis regimen**	**Chemical**	279,719	331	1·18	[1·06 to 1·32]	507,447	537	1·06	[0·97 to 1·15]	1,628,867	970	0·60	[0·56 to 0·63]
	**Non chemical**	30,000	43	1·43	[1·04 to 1·93]	57,691	75	1·30	[1·02 to 1·63]	278,679	173	0·62	[0·53 to 0·72]
**Acetabulum bonegraft**	**No**	296,978	352	1·19	[1·06 to 1·32]	541,929	585	1·08	[0·99 to 1·17]	1,828,100	1097	0·60	[0·57 to 0·64]
	**Yes**	12,741	22	1·73	[1·08 to 2·61]	23,210	27	1·16	[0·77 to 1·69]	79,446	46	0·58	[0·42 to 0·77]
**Femur bonegraft**	**No**	307,322	368	1·20	[1·08 to 1·33]	560,832	601	1·07	[0·99 to 1·16]	1,891,923	1129	0·60	[0·56 to 0·63]
	**Yes**	2,396	6	2·50	[0·92 to 5·45]	4,307	11	2·55	[1·28 to 4·57]	15,623	14	0·90	[0·49 to 1·50]
**Intra operative event**	**No**	306,119	365	1·19	[1·07 to 1·32]	558,612	601	1·08	[0·99 to 1·17]	1,887,934	1132	0·60	[0·57 to 0·64]
	**Yes**	3,600	9	2·50	[1·14 to 4·75]	6,527	11	1·69	[0·84 to 3·02]	19,612	11	0·56	[0·28 to 1·00]
**Place of surgery**	**England**	292,280	357	1·22	[1·10 to 1·35]	533,181	565	1·06	[0·97 to 1·15]	1,801,681	1,075	0·60	[0·56 to 0·63]
	**Wales**	17,438	17	0·97	[0·57 to 1·56]	31,958	47	1·47	[1·08 to 1·96]	105,865	68	0·64	[0·50 to 0·81]
**Funding**	**NHS**	251,631	294	1·17	[1·04 to 1·31]	456,129	504	1·10	[1·01 to 1·21]	1,438,428	886	0·62	[0·58 to 0·66]
	**Independent**	45,234	58	1·28	[0·97 to 1·66]	84,019	70	0·83	[0·65 to 1·05]	327,119	154	0·47	[0·40 to 0·55]
	**Unspecified**	12,854	22	1·71	[1·07 to 2·59]	24,990	38	1·52	[1·08 to 2·09]	142,000	103	0·73	[0·59 to 0·88]
**Grade operating surgeon**	**Consultant**	261,858	310	1·18	[1·06 to 1·32]	477,609	499	1·04	[0·96 to 1·14]	1,602,405	965	0·60	[0·56 to 0·64]
	**Other**	47,861	64	1·34	[1·03 to 1·71]	87,529	113	1·29	[1·06 to 1·55]	305,141	178	0·58	[0·50 to 0·68]
**Consultant involvement**	**Operating**	261,858	310	1·18	[1·06 to 1·32]	477,609	499	1·04	[0·96 to 1·14]	1,602,405	965	0·60	[0·56 to 0·64]
	**Assisting**	16,484	28	1·70	[1·13 to 2·45]	29,684	42	1·41	[1·02 to 1·91]	95,920	52	0·54	[0·40 to 0·71]
	**Not involved**	31,377	36	1·15	[0·80 to 1·59]	57,845	71	1·23	[0·96 to 1·55]	209,220	126	0·60	[0·50 to 0·72]
**Total volume Operating surgeon**	**≤28**	81,590	110	1·35	[1·11 to 1·62]	151,504	195	1·29	[1·11 to 1·48]	615,137	363	0·59	[0·53 to 0·65]
	**]28–63]**	78,630	97	1·23	[1·00 to 1·50]	144,369	163	1·13	[0·96 to 1·32]	497,021	330	0·66	[0·59 to 0·74]
	**]63–114]**	76,498	92	1·20	[0·97 to 1·47]	138,218	135	0·98	[0·82 to 1·16]	428,485	236	0·55	[0·48 to 0·63]
	**>114**	73,001	75	1·03	[0·81 to 1·29]	131,048	119	0·91	[0·75 to 1·09]	366,903	214	0·58	[0·51 to 0·67]
**Total volume Surgeon in charge**	**≤41**	82,284	91	1·11	[0·89 to 1·36]	153,422	186	1·21	[1·04 to 1·40]	632,674	382	0·60	[0·54 to 0·67]
	**]42–84]**	78,551	104	1·32	[1·08 to 1·60]	143,267	146	1·02	[0·86 to 1·20]	481,722	289	0·60	[0·53 to 0·67]
	**]84–148]**	75,708	96	1·27	[1·03 to 1·55]	137,668	159	1·15	[0·98 to 1·35]	429,766	229	0·53	[0·47 to 0·61]
	**>148**	73,176	83	1·13	[0·90 to 1·41]	130,781	121	0·93	[0·77 to 1·11]	363,385	243	0·67	[0·59 to 0·76]
**Total volume Hospital**	**≤143**	79,703	111	1·39	[1·15 to 1·68]	149,344	167	1·12	[0·96 to 1·30]	652,640	387	0·59	[0·54 to 0·66]
	**]143–256]**	78,616	81	1·03	[0·82 to 1·28]	144,023	158	1·10	[0·93 to 1·28]	499,247	298	0·60	[0·53 to 0·67]

**]256–406]**	76,705	81	1·06	[0·84 to 1·31]	137,925	143	1·04	[0·87 to 1·22]	381,901	206	0·54	[0·47 to 0·62]
**>406**	74,694	101	1·35	[1·10 to 1·64]	133,847	144	1·08	[0·91 to 1·27]	373,759	252	0·67	[0·59 to 0·76]

Incidence rate per 1,000 person-years, 95%CI: 95% confidence interval

1. BMI=Body Mass Index
2. ASA=American Society of Anaesthesiologists grade.
3. THR=Total Hip Replacement
4. MoP=metal on polyethylene; MoM=metal on metal; CoP=ceramic on polyethylene; CoC=ceramic on ceramic; CoM=ceramic on metal

Appendix Table 2: Incidence rate ratios of revision for PJI and 95% credible intervals for patient characteristics

		Total RR	95%CI	P-value	Adj. p-value	≤3mths RR	95%CI	P-value	Adj. p-value	3–6mths RR	95%CI	P-value	Adj. p-value	6–12mths RR	95%CI	P-value	Adj. p-value	12–24mths RR	95%CI	P-value	Adj. p-value	≥24mths RR	95%CI	P-value	Adj. p-value
**Sex**	**Male**	1·68	[1·56 to 1·81]	<0·0001	<0·0001	1·72	[1·39 to 2·10]	<0·0001	<0·0001	2·59	[1·93 to 3·44]	<0·0001	<0·0001	1·93	[1·57 to 2·37]	<0·0001	<0·0001	1·73	[1·47 to 2·03]	<0·0001	<0·0001	1·44	[1·28 to 1·62]	<0·0001	<0·0001
	**Female**	Ref				Ref				Ref				Ref				Ref				Ref			
**Age**	**≥80**	0·66	[0·56 to 0·76]	<0·0001	<0·0001	1·42	[0·98 to 1·99]	0.07	0·20	0·63	[0·32 to 1·10]	0.11	0·26	0·41	[0·25 to 0·63]	<0·0001	<0·0001	0·68	[0·49 to 0·91]	0.01	0·05	0·48	[0·37 to 0·61]	<0·0001	<0·0001
	**[70–79]**	0·73	[0·66 to 0·81]	<0·0001	<0·0001	0·97	[0·70 to 1·30]	0.77	0·86	1·26	[0·84 to 1·82]	0.29	0·49	0·67	[0·49 to 0·89]	0.006	0·03	0·69	[0·55 to 0·86]	0.001	0·007	0·64	[0·54 to 0·74]	<0·0001	<0·0001
	**[60–69]**	0·90	[0·81 to 0·99]	0.03	0·11	1·14	[0·84 to 1·52]	0.41	0·63	1·05	[0·69 to 1·54]	0.89	0·95	1·11	[0·85 to 1·43]	0.48	0·68	0·93	[0·75 to 1·14]	0.47	0·67	0·76	[0·66 to 0·88]	<0·0001	<0·0001
	**<60**	Ref				Ref				Ref				Ref				Ref				Ref			
**Ethn-city**	**Black African origin**	0·80	[0·41 to 1·33]	0.37	0·58					0·99	[0·03 to 3·68]	0.64	0·80	0·49	[0·01 to 1·82]	0.31	0·51	0·57	[0·07 to 1·59]	0.29	0·49	1·23	[0·53 to 2·26]	0.70	0·83
	**Other and mixed**	0·88	[0·48 to 1·40]	0.51	0·69					3·62	[0·98 to 8·04]	0.06	0·11	1·74	[0·47 to 3·86]	0.43	0·65	0·27	[0·01 to 1·00]	0.14	0·30	0·75	[0·25 to 1·56]	0.41	0·63
	**South Asian**	0·70	[0·25 to 1·36]	0.26	0·47	0·98	[0·02 to 3·62]	0.64	0·79	1·70	[0·04 to 6·36]	0.97	0·99					1·02	[0·12 to 2·87]	0.76	0·85	0·56	[0·07 to 1·57]	0.29	0·49
	**Unclear**	0·25	[0·16 to 0·36]	<0·0001	<0·0001	0·43	[0·14 to 0·90]	0.05	0·15	0·31	[0·04 to 0·87]	0.05	0·21	0·23	[0·05 to 0·55]	0.009	0·04	0·23	[0·08 to 0·48]	0.001	0·007	0·21	[0·10 to 0·36]	<0·0001	<0·0001
	**White**	Ref				Ref				Ref				Ref				Ref				Ref			
**BMI[1]**	**≥30**	1·92	[1·72 to 2·15]	<0·0001	<0·0001	2·99	[2·12 to 4·13]	<0·0001	<0·0001	2·41	[1·51 to 3·75]	<0·0001	<0·0001	1·54	[1·14 to 2·04]	0.02	0·07	1·99	[1·56 to 2·53]	<0·0001	<0·0001	1·72	[1·45 to 2·03]	<0·0001	<0·0001
	**[25–29·9]**	1·25	[1·11 to 1·40]	0.001	0·007	1·57	[1·09 to 2·22]	0.04	0·14	1·67	[1·03 to 2·61]	0.05	0·18	0·88	[0·64 to 1·19]	0.39	0·61	1·31	[1·01 to 1·68]	0.05	0·25	1·25	[1·05 to 1·49]	0.03	0·12
	**<25**	Ref				Ref				Ref				Ref				Ref				Ref			
**ASA[2]**	**3–5**	1·63	[1·42 to 1·87]	<0·0001	<0·0001	2·40	[1·57 to 3·55]	<0·0001	<0·0001	2·38	[1·26 to 4·15]	0.007	0·03	2·43	[1·65 to 3·48]	<0·0001	<0·0001	1·64	[1·21 to 2·16]	0.001	0·007	1·12	[0·89 to 1·37]	0.34	0·55
	**2**	1·26	[1·14 to 1·40]	<0·0001	<0·0001	1·64	[1·14 to 2·32]	0.008	0·04	2·30	[1·38 to 3·73]	0.002	0·01	1·49	[1·08 to 2·03]	0.02	0·06	1·25	[0·99 to 1·57]	0.07	0·19	1·04	[0·89 to 1·20]	0.65	0·80
	**1**	Ref				Ref				Ref				Ref				Ref				Ref			
**Chronic Pulmonary Disease**		1·22	[1·08 to 1·37]	0.02	0·01	1·41	[1·03 to 1·86]	0.03	0·10	1·03	[0·62 to 1·55]	0.99	0·99	1·28	[0·92 to 1·71]	0.13	0·29	1·32	[1·03 to 1·65]	0.03	0·09	0·96	[0·77 to 1·18]	0.67	0·81
**Diabetes**		1·35	[1·18 to 1·54]	<0·0001	<0·0001	1·53	[1·09 to 2·07]	0.01	0·05	1·60	[1·01 to 2·35]	0.04	0·13	1·52	[1·07 to 2·07]	0.02	0·06	1·32	[0·99 to 1·71]	0.06	0·16	1·00	[0·78 to 1·27]	0.98	0·99
**Dementia**		1·85	[0·89 to 3·18]	0.08	0·22	3·78	[1·21 to 7·81]	0.01	0·05	1·80	[0·04 to 6·68]	0.99	0·99	2·32	[0·28 to 6·51]	0.48	0·68	0·66	[0·02 to 2·45]	0.44	0·65	0·60	[0·02 to 2·22]	0.40	0·61
**Liver disease**		2·35	[1·66 to 3·17]	<0·0001	<0·0001	2·24	[0·81 to 4·38]	0.10	0·24	1·39	[0·17 to 3·88]	0.95	0·98	2·52	[1·01 to 4·75]	0.03	0·11	1·90	[0·82 to 3·45]	0.11	0·27	2·43	[1·35 to 3·82]	0.001	0·007
**Congestive Heart Failure**		1·45	[1·09 to 1·86]	0.007	0·03	1·65	[0·86 to 2·71]	0.12	0·27					2·34	[1·26 to 3·77]	0.004	0·02	1·27	[0·67 to 2·07]	0.49	0·68	1·27	[0·74 to 1·93]	0.39	0·61
**Rheumatologic Disease**		1·37	[1·12 to 1·64]	0.001	0·007	1·49	[0·88 to 2·28]	0.12	0·28	2·28	[1·22 to 3·72]	0.006	0·03	1·6	[0·94 to 2·43]	0.07	0·20	1·14	[0·71 to 1·67]	0.62	0·78	1·12	[0·79 to 1·51]	0.55	0·73
**Cancer**	**Non-metastatic**	1·12	[0·89 to 1·37]	0.35	0·56	1·01	[0·53 to 1·66]	0.92	0·97	1·14	[0·49 to 2·11]	0.86	0·93	0·95	[0·47 to 1·61]	0.75	0·85	0·95	[0·56 to 1·46]	0.75	0·85	1·22	[0·84 to 1·67]	0.29	0·49
	**Metastatic**	1·81	[1·07 to 2·74]	0.02	0·07	0·81	[0·10 to 2·26]	0.55	0·73	1·85	[0·22 to 5·20]	0.67	0·81	2·21	[0·60 to 4·86]	0.22	0·42	1·53	[0·42 to 3·38]	0.58	0·75	1·82	[0·66 to 3·55]	0.23	0·42
	**None**	Ref				Ref				Ref				Ref				Ref				Ref			
**Cerebrovascular Disease**		0·94	[0·65 to 1·27]	0.65	0·80	1·45	[0·68 to 2·52]	0.34	0·55	0·87	[0·18 to 2·11]	0.61	0·78	1·22	[0·49 to 2·31]	0.75	0·85	0·62	[0·23 to 1·22]	0.19	0·38	0·71	[0·34 to 1·21]	0.22	0·42
**Myocardial Infarction**		0·99	[0·75 to 1·26]	0.89	0·95	1·17	[0·60 to 1·95]	0.70	0·83	0·53	[0·11 to 1·28]	0.19	0·39	0·65	[0·23 to 1·27]	0.22	0·42	1·14	[0·64 to 1·78]	0.71	0·83	1·00	[0·63 to 1·46]	0.93	0·97
**Paraplegia and Hemiplegia**		1·62	[0·88 to 2·57]	0.10	0·25	0·7	[0·02 to 2·60]	0.47	0·67	2·78	[0·33 to 7·80]	0.35	0·56	1·51	[0·18 to 4·19]	0.86	0·93	2·32	[0·74 to 4·81]	0.12	0·28	1·18	[0·32 to 2·58]	0.95	0·98
**Peptic Ulcer Disease**		1·12	[0·76 to 1·54]	0.59	0·76	1·16	[0·37 to 2·40]	0.92	0·97	0·88	[0·11 to 2·49]	0.62	0·78	1·53	[0·55 to 2·99]	0.43	0·65	0·76	[0·24 to 1·55]	0.42	0·63	1·20	[0·66 to 1·92]	0.58	0·76
**Peripheral Vascular Disease**		1·19	[0·87 to 1·55]	0.28	0·49	1·25	[0·57 to 2·23]	0.63	0·79	1·01	[0·27 to 2·24]	0.82	0·89	1·76	[0·86 to 2·97]	0.10	0·25	0·87	[0·40 to 1·54]	0.58	0·75	1·00	[0·54 to 1·60]	0.89	0·95
**Renal Disease**		1·12	[0·84 to 1·45]	0.45	0·66	1·21	[0·65 to 1·95]	0.59	0·76	1·70	[0·76 to 3·05]	0.18	0·38	0·93	[0·40 to 1·70]	0.71	0·83	0·62	[0·28 to 1·10]	0.13	0·28	0·97	[0·53 to 1·55]	0.82	0·89

RR: Rate ratio adjusted for age to sex to Body Mass Index and ASA grade; CI: 95% Confidence Interval; Adj. p-value: q-value or adjusted p-value.

1. Body Mass Index:Kg/m^2
2. American Society of Anaesthesiologists scale of surgical fitness.

Appendix Table 3: Rate ratios of revision for PJI and 95%credible intervals for surgical characteristics

		Total				≤3mths				3–6mths				6–12mths				12–24mths				≥24mths			
		**RR**	**95%CI**	**P-value**	**Adj. p-value**	**RR**	**95%CI**	**P-value**	**Adj. p-value**	**RR**	**95%CI**	**P-value**	**Adj. p-value**	**RR**	**95%CI**	**P-value**	**Adj. p-value**	**RR**	**95%CI**	**P-value**	**Adj. p-value**	**RR**	**95%CI**	**P-value**	**Adj. p-value**
**Osteoarthritis**		0·69	[0·60 to 0·79]	<0·0001	<0·0001	0·67	[0·47 to 0·94]	0.02	0·06	0·49	[0·32 to 0·75]	0.001	0·007	0·57	[0·41 to 0·77]	<0·0001	<0·0001	0·79	[0·59 to 1·05]	0.10	0·25	0·80	[0·66 to 1·03]	0.09	0·25
**Fractured neck of femur**		1·82	[1·40 to 2·30]		<0·0001	3·00	[1·82 to 4·49]	<0·0001	<0·0001	2·07	[0·81 to 3·94]	0.11	0·26	1·38	[0·62 to 2·46]	0.46	0·67	1·32	[0·70 to 2·14]	0.41	0·63	1·30	[0·71 to 2·00]	0.45	0·66
**Previous hip infection**		6·69	[4·18 to 9·80]	<0·0001	<0·0001	6·71	[1·38 to 16·31]	0.006	0·03	23·30	[7·38 to 48·63]	<0·0001	<0·0001	6·45	[1·31 to 15·75]	0.008	0·04	6·92	[2·25 to 14·28]	<0·0001	<0·0001	4·30	[1·56 to 8·40]	0.001	0·007
**Avascular necrosis**		1·42	[1·16 to 1·71]	<0·0001	<0·0001	1·53	[0·84 to 2·44]	0.16	0·33	1·16	[0·42 to 2·30]	0.89	0·95	1·73	[1·02 to 2·66]	0.03	0·11	1·42	[0·90 to 2·07]	0.12	0·28	1·40	[0·99 to 1·81]	0.05	0·16
**Dysplasia/ Congenital dislocation**		0·64	[0·42 to 0·90]	0.02	0·06	0·43	[0·05 to 1·21]	0.16	0·34	0·89	[0·11 to 2·50]	0.62	0·78	0·73	[0·20 to 1·61]	0.40	0·62	0·52	[0·17 to 1·08]	0.12	0·27	0·70	[0·38 to 1·09]	0.13	0·28
**Inflammatory arthropathy**		0·90	[0·64 to 1·21]	0.47	0·67	0·35	[0·04 to 1·00]	0.11	0·26	1·81	[0·58 to 3·79]	0.31	0·51	1·49	[0·67 to 2·64]	0.34	0·55	0·83	[0·36 to 1·51]	0.50	0·69	0·80	[0·48 to 1·31]	0.42	0·63
**Surgical approach**	**Lateral**	1·32	[1·21 to 1·43]	<0·0001	<0·0001	1·08	[0·86 to 1·35]	0.54	0·73	1·56	[1·16 to 2·06]	0.003	0·02	1·51	[1·21 to 1·87]	<0·0001	<0·0001	1·53	[1·28 to 1·81]	<0·0001	<0·0001	1·32	[1·16 to 1·50]	<0·0001	<0·0001
	**Other**	1·48	[1·22 to 1·77]		<0·0001	1·33	[0·79 to 2·03]		0·49	1·20	[0·53 to 2·18]		0·85	1·39	[0·80 to 2·16]		0·44	1·32	[0·85 to 1·89]		0·41	1·60	[1·15 to 2·06]		0·03
	**Posterior**	Ref				Ref				Ref				Ref				Ref				Ref			
**Procedure[1]**	**Resurfac·**	0·56	[0·46 to 0·67]	<0·0001	<0·0001	0·68	[0·30 to 1·25]	0.21	0·41	0·32	[0·11 to 0·67]	0.007	0·03	0·39	[0·20 to 0·65]	0.001	0·007	0·53	[0·35 to 0·76]	0.001	0·007	0·70	[0·51 to 0·83]	0.001	0·007
	**THR uncement·**	0·92	[0·83 to 1·01]	0.09	0·23	1·56	[1·17 to 2·05]	0.002	0·01	0·67	[0·48 to 0·93]	0.02	0·06	0·66	[0·51 to 0·85]	0.001	0·007	0·79	[0·64 to 0·96]	0.02	0·06	0·90	[0·79 to 1·06]	0.23	0·43
	**THR other**	0·87	[0·77 to 0·98]	0.02	0·07	1·60	[1·17 to 2·14]	0.003	0·02	0·61	[0·39 to 0·90]	0.02	0·07	0·95	[0·70 to 1·25]	0.68	0·81	0·60	[0·45 to 0·77]	<0·0001	<0·0001	0·80	[0·65 to 0·95]	0.01	0·06
	**THR cemented**	Ref				Ref				Ref				Ref				Ref				Ref			
**Type of bearing[2]**	**MoM**	1·07	[0·95 to 1·21]	0.27	0·47	0·72	[0·45 to 1·08]	0.12	0·27	0·42	[0·21 to 0·72]	0.004	0·02	0·73	[0·49 to 1·02]	0.07	0·21	1·21	[0·93 to 1·54]	0.16	0·34	1·40	[1·21 to 1·69]	<0·0001	<0·0001
	**CoP**	0·82	[0·71 to 0·95]	0.007	0·03	1·08	[0·74 to 1·50]	0.74	0·84	0·61	[0·34 to 0·96]	0.04	0·14	0·97	[0·69 to 1·30]	0.77	0·86	0·72	[0·53 to 0·96]	0.03	0·10	0·70	[0·52 to 0·85]	0.001	0·007
	**CoC**	0·76	[0·66 to 0·86]	<0·0001	<0·0001	1·21	[0·87 to 1·63]	0.27	0·47	0·78	[0·50 to 1·16]	0.22	0·42	0·64	[0·45 to 0·87]	0.007	0·03	0·77	[0·59 to 0·99]	0.04	0·14	0·55	[0·43 to 0·69]	<0·0001	<0·0001
	**CoM**	1·88	[1·13 to 2·83]	0.01	0·05	0·79	[0·02 to 2·95]	0.52	0·71	1·11	[0·03 to 4·16]	0.71	0·83	1·86	[0·38 to 4·55]	0.49	0·68	1·45	[0·39 to 3·25]	0.66	0·80	2·60	[1·26 to 4·38]	0.005	0·03
	**Other**	0·98	[0·74 to 1·25]	0.81	0·89	1·81	[0·91 to 3·05]	0.08	0·21	1·11	[0·36 to 2·33]	0.99	0·99	1·33	[0·63 to 2·32]	0.49	0·68	0·92	[0·46 to 1·56]	0.68	0·82	0·80	[0·49 to 1·18]	0.27	0·47
	**MoP**	Ref				Ref				Ref				Ref				Ref				Ref			
**General Anaesthesia**		1·08	[0·99 to 1·18]	0.07	0·20	1·05	[0·83 to 1·30]	0.74	0·84	1·27	[0·95 to 1·67]	0.11	0·26	1·20	[0·97 to 1·48]	0.10	0·25	1·00	[0·84 to 1·18]	0.99	0·99	1·10	[0·97 to 1·25]	0.12	0·28
**Nerve Block Anaesthesia**		0·97	[0·85 to 1·10]	0.63	0·79	1·14	[0·80 to 1·55]	0.50	0·69	1·15	[0·72 to 1·71]	0.60	0·76	0·81	[0·54 to 1·14]	0.23	0·42	1·02	[0·77 to 1·32]	0.92	0·97	0·90	[0·76 to 1·13]	0.48	0·68
**Epidural Anaesthesia**		0·96	[0·84 to 1·08]	0.46	0·67	0·92	[0·61 to 1·31]	0.61	0·78	1·20	[0·72 to 1·82]	0.52	0·71	1·33	[0·94 to 1·80]	0.10	0·25	1·05	[0·78 to 1·35]	0.80	0·89	1·00	[0·80 to 1·16]	0.70	0·83
**Spinal Anaesthesia**		0·93	[0·86 to 1·01]	0.08	0·21	0·88	[0·71 to 1·10]	0.25	0·46	0·77	[0·57 to 1·01]	0.052	0·16	0·95	[0·76 to 1·16]	0.57	0·75	0·96	[0·81 to 1·13]	0.59	0·76	0·90	[0·79 to 1·01]	0.08	0·21
**Thrombop· regimen**	**Not chemical**	0·96	[0·84 to 1·09]	0.55	0·73	0·85	[0·54 to 1·24]	0.38	0·59	1·17	[0·71 to 1·76]	0.57	0·75	1·25	[0·87 to 1·70]	0.23	0·43	1·31	[0·99 to 1·68]	0.054	0·16	1·00	[0·83 to 1·20]	0.99	0·99
	**Chemical**	Ref				Ref				Ref				Ref				Ref				Ref			
**Acetabulum bone graft**		1·08	[0·89 to 1·29]	0.45	0·66	0·75	[0·37 to 1·27]	0.28	0·49	1·41	[0·69 to 2·40]	0.35	0·56	1·50	[0·92 to 2·22]	0.09	0·23	1·10	[0·71 to 1·57]	0.71	0·83	1·00	[0·71 to 1·29]	0.82	0·89
**Femur bone graft**		1·84	[1·29 to 2·50]	<0·0001	<0·0001	2·35	[0·84 to 4·66]	0.08	0·22	0·66	[0·02 to 2·47]	0.44	0·66	2·09	[0·76 to 4·12]	0.14	0·30	2·46	[1·21 to 4·17]	0.007	0·03	1·50	[0·81 to 2·40]	0.19	0·38
**Intra operative event**		1·48	[1·06 to 1·98]	0.02	0·07	1·93	[0·82 to 3·52]	0.11	0·27	1·40	[0·29 to 3·40]	0.80	0·89	2·08	[0·93 to 3·72]	0.06	0·17	1·62	[0·80 to 2·73]	0.17	0·35	1·00	[0·48 to 1·64]	0.82	0·89

RR: Rate ratio adjusted for age to sex to Body Mass Index and ASA grade; CI: 95% Confidence Interval; Adj. p-value: q-value or adjusted p-value.

1. THR: Total hip replacement
2. MoP=metal on polyethylene; MoM=metal on metal; CoP=ceramic on polyethylene; CoC=ceramic on ceramic; CoM=Ceramic on metal; Other=a combination of unclassifiable components

Appendix Table 4: Rate ratios of revision for PJI and 95%credible intervals for health system characteristics

		Total				≤3mths				3–6mths				6–12mths				12–24mths				≥24mths			
		**RR**	**95%CI**	**P-value**	**Adj. p-value**	**RR**	**95%CI**	**P-value**	**Adj. p-value**	**RR**	**95%CI**	**P-value**	**Adj. p-value**	**RR**	**95%CI**	**P-value**	**Adj. p-value**	**RR**	**95%CI**	**P-value**	**Adj. p-value**	**RR**	**95%CI**	**P-value**	**Adj. p-value**
**Place of surgery**	**Wales**	1·04	[0·80 to 1·33]	0.80	0·88	1·07	[0·54 to 1·88]	0.95	0·98	0·91	[0·43 to 1·60]	0.65	0·80	0·76	[0·41 to 1·24]	0.26	0·47	1·37	[0·89 to 1·98]	0.15	0·32	1·03	[0·73 to 1·39]	0.95	0·98
	**England**	Ref				Ref				Ref				Ref				Ref				Ref			
**Funding**	**Independent**	0·88	[0·77 to 0·99]	0.04	0·13	0·94	[0·64 to 1·31]	0.65	0·80	1·1	[0·70 to 1·60]	0.73	0·84	1·19	[0·87 to 1·58]	0.29	0·49	0·83	[0·63 to 1·07]	0.15	0·33	0·81	[0·67 to 0·97]	0.03	0·10
	**Unspecified**	0·98	[0·83 to 1·15]	0.81	0·89	0·39	[0·15 to 0·75]	0.01	0·06	1·1	[0·49 to 1·98]	0.92	0·97	1·49	[0·91 to 2·24]	0.10	0·25	1·37	[0·95 to 1·88]	0.09	0·23	1·19	[0·95 to 1·46]	0.13	0·29
	**NHS**	Ref				Ref				Ref				Ref				Ref				Ref			
**Grade operating surgeon**	**Other**	1·08	[0·96 to 1·20]	0.21	0·41	1·03	[0·76 to 1·36]	0.89	0·95	1·1	[0·73 to 1·56]	0.69	0·82	1·18	[0·88 to 1·54]	0.27	0·48	1·25	[1·00 to 1·54]	0.05	0·15	1·02	[0·85 to 1·20]	0.87	0·94
	**Consultant**	Ref				Ref				Ref				Ref				Ref				Ref			
**Consultant involved**	**None involved**	1·04	[0·91 to 1·19]	0.15	0·73	0·94	[0·64 to 1·32]	0.47	0·81	0·94	[0·55 to 1·46]	0.67	0·84	1·02	[0·69 to 1·43]	0.98	0·99	1·25	[0·95 to 1·60]	0.12	0·27	1·07	[0·87 to 1·30]	0.55	0·73
	**Assisting**	1·13	[0·95 to 1·33]	0.15	0·32	1·19	[0·75 to 1·74]	0.47	0·67	1·38	[0·75 to 2·22]	0.31	0·51	1·47	[0·95 to 2·11]	0.07	0·21	1·26	[0·90 to 1·71]	0.18	0·37	0·92	[0·68 to 1·20]	0.53	0·72
	**Operating**	Ref				Ref				Ref				Ref				Ref				Ref			
**Total volume Operating surgeon**	**>114**	0·87	[0·77 to 0·98]	0.02	0·07	1·04	[0·75 to 1·39]	0.87	0·94	0·64	[0·41 to 0·94]	0.03	0·10	0·72	[0·53 to 0·97]	0.03	0·11	0·69	[0·54 to 0·87]	0.002	0·01	0·88	[0·73 to 1·05]	0.17	0·35
	**]63–114]**	0·86	[0·77 to 0·96]	0.007	0·03	0·78	[0·56 to 1·04]	0.09	0·23	0·77	[0·51 to 1·11]	0.16	0·33	0·85	[0·63 to 1·11]	0.22	0·42	0·74	[0·59 to 0·92]	0.008	0·04	0·86	[0·73 to 1·02]	0.08	0·22
	**]28–63]**	1·02	[0·92 to 1·13]	0.68	0·81	1·04	[0·78 to 1·35]	0.86	0·93	0·97	[0·67 to 1·37]	0.80	0·89	0·89	[0·67 to 1·16]	0.36	0·57	0·88	[0·71 to 1·08]	0.21	0·41	1·08	[0·93 to 1·25]	0.33	0·54
	**≤28**	Ref				Ref				Ref				Ref				Ref				Ref			
**Tot volume Surgeon in charge**	**>148**	1·00	[0·89 to 1·12]	0.97	0·99	1·13	[0·83 to 1·50]	0.48	0·68	0·69	[0·45 to 1·02]	0.06	0·19	1·01	[0·73 to 1·36]	0.98	0·99	0·75	[0·58 to 0·94]	0.02	0·06	1·01	[0·84 to 1·19]	0.99	0·99
	**]84–148]**	0·93	[0·83 to 1·04]	0.20	0·39	0·85	[0·63 to 1·13]	0.27	0·47	0·72	[0·48 to 1·05]	0.09	0·23	1·13	[0·83 to 1·50]	0.46	0·67	0·92	[0·73 to 1·14]	0.42	0·63	0·83	[0·70 to 0·98]	0.03	0·10
	**]41–84]**	0·97	[0·87 to 1·07]	0.54	0·73	0·84	[0·62 to 1·11]	0.21	0·41	0·95	[0·65 to 1·33]	0.69	0·82	1·18	[0·88 to 1·56]	0.28	0·49	0·83	[0·66 to 1·03]	0.09	0·23	0·96	[0·82 to 1·12]	0.58	0·75
	**≤41**	Ref				Ref				Ref				Ref				Ref				Ref			
**Total volume Hospital**	**>406**	1·28	[1·10 to 1·48]	0.001	0·007	1·91	[1·26 to 2·81]	0.002	0·01	1·19	[0·79 to 1·74]	0.44	0·65	1·01	[0·73 to 1·37]	0.98	0·99	0·88	[0·66 to 1·15]	0.34	0·55	1·12	[0·90 to 1·37]	0.33	0·54
	**]256–406]**	1·03	[0·90 to 1·16]	0.70	0·83	1·57	[1·09 to 2·22]	0.02	0·06	0·9	[0·59 to 1·31]	0.55	0·73	0·74	[0·54 to 0·99]	0.05	0·15	0·86	[0·67 to 1·09]	0.22	0·42	0·89	[0·73 to 1·06]	0.19	0·39
	**]143–256]**	1·01	[0·90 to 1·13]	0.89	0·95	1·23	[0·86 to 1·72]	0.28	0·49	0·76	[0·49 to 1·13]	0.17	0·35	0·72	[0·53 to 0·96]	0.03	0·10	0·95	[0·75 to 1·19]	0.63	0·79	1·01	[0·86 to 1·18]	0.90	0·95
	**≤143**	Ref				Ref				Ref				Ref				Ref				Ref			

RR: Rate ratio adjusted for age to sex to Body Mass Index and ASA grade; CI: 95% Confidence Interval; Adj. p-value: q-value or adjusted p-value.

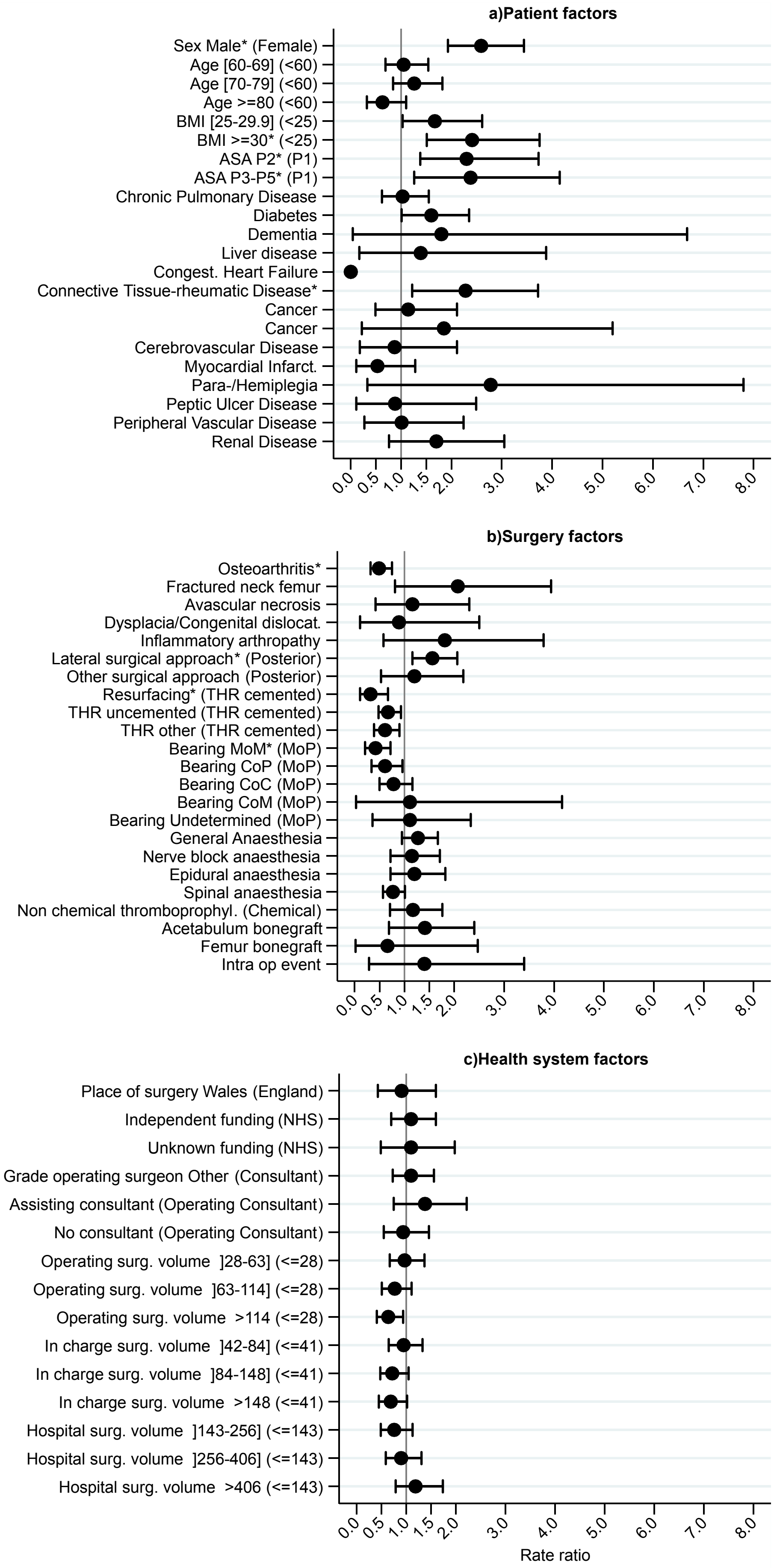

Appendix Figure 1: Risk factors of revision for prosthetic joint infection for the 3-6 postoperative months
Reference category in parentheses. BMI=body-mass index. ASA=American Society of Anaesthesiologists. THR=total hip replacement. MoM=metal-on-metal. MoP=metal-on-polyethylene. CoP=ceramic-on-polyethylene. CoC=ceramic-on-ceramic. CoM=metal-on-ceramic. Undetermined =a combination of unclassifiable components *Adjusted p value<0.05

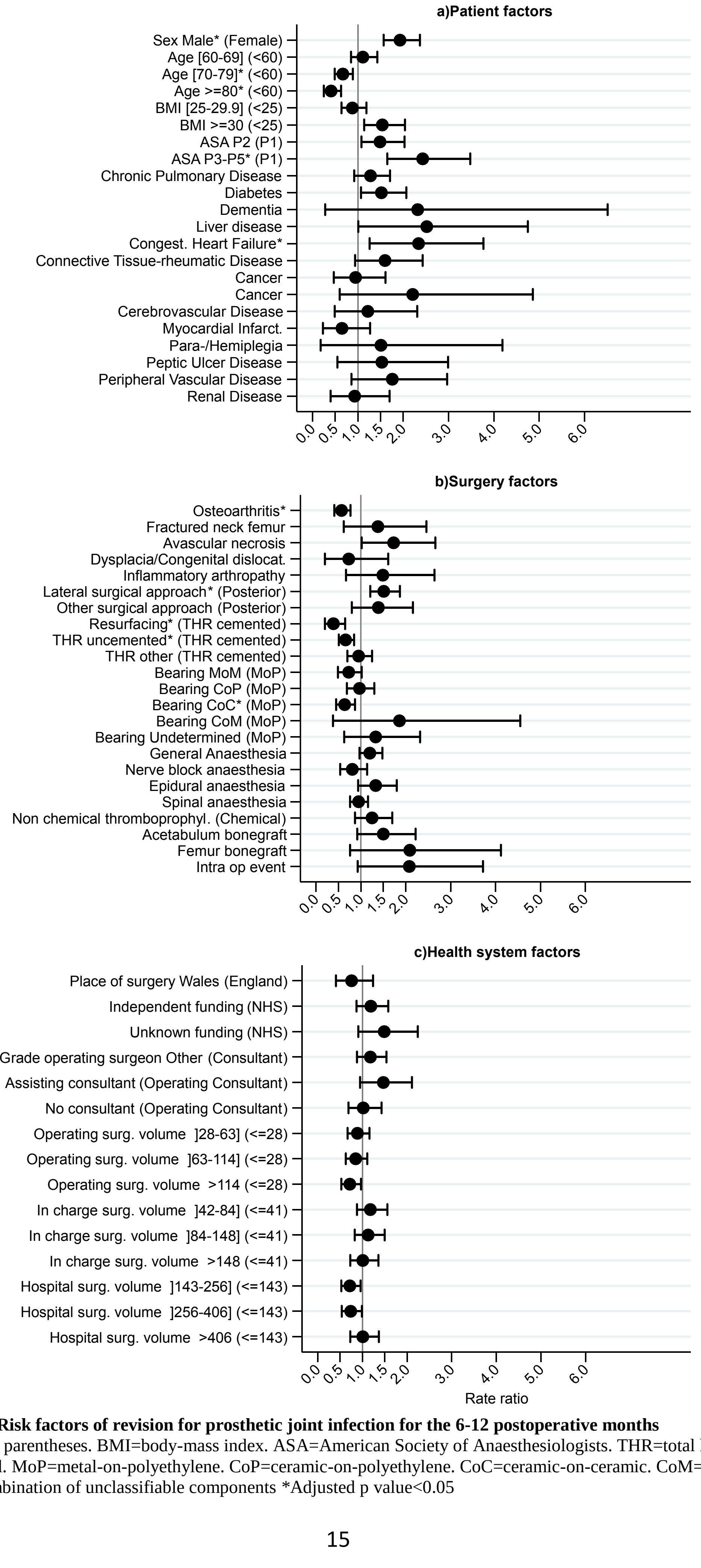

Appendix Figure 2: Risk factors of revision for prosthetic joint infection for the 6-12 postoperative months
Reference category in parentheses. BMI=body-mass index. ASA=American Society of Anaesthesiologists. THR=total hip replacement. MoM=metal-on-metal. MoP=metal-on-polyethylene. CoP=ceramic-on-polyethylene. CoC=ceramic-on-ceramic. CoM=metal-on-ceramic. Undetermined =a combination of unclassifiable components *Adjusted p value<0.05

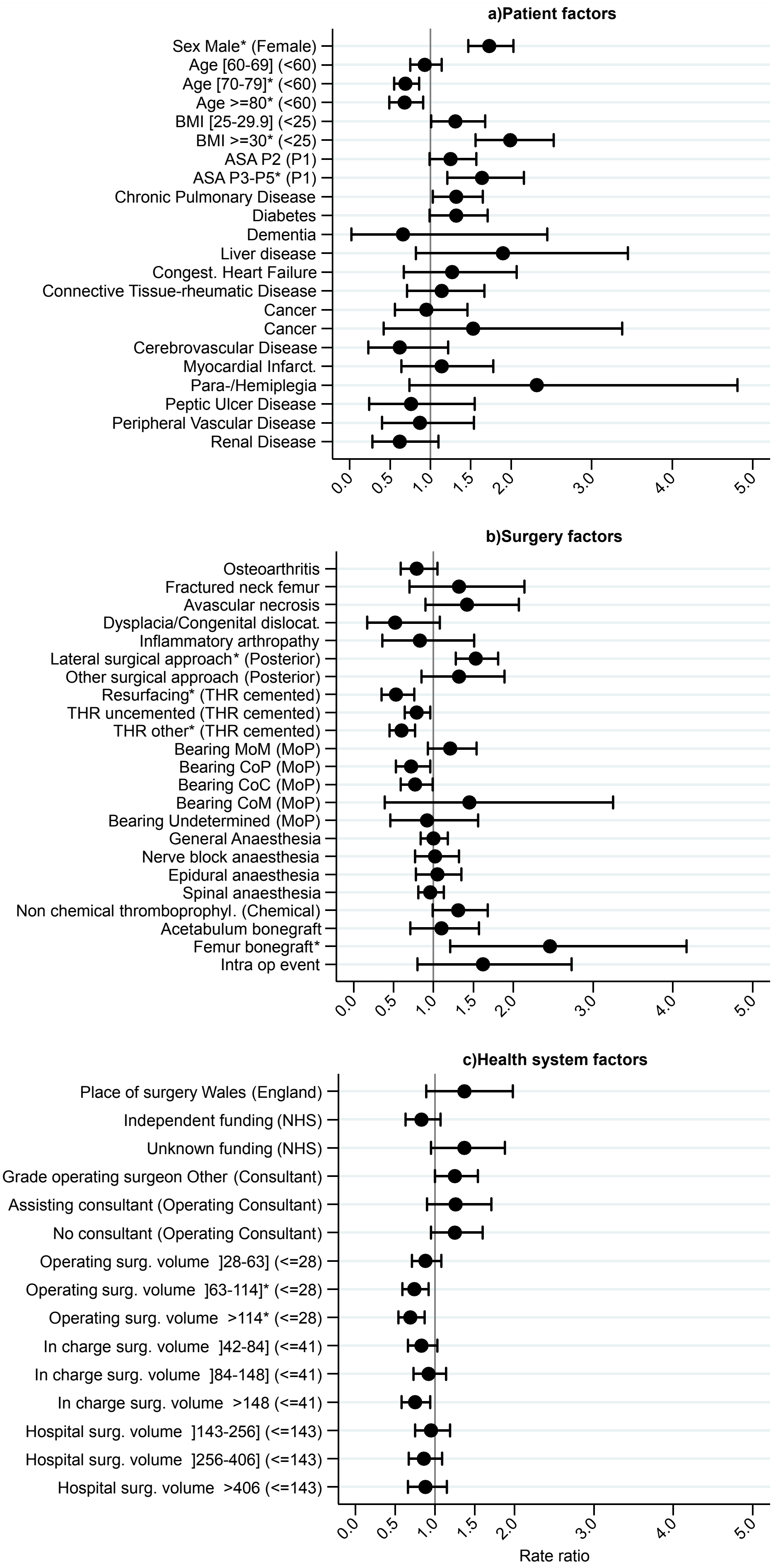

Appendix Figure 3: Risk factors of revision for prosthetic joint infection for the 12-24 postoperative months

Reference category in parentheses. BMI=body-mass index. ASA=American Society of Anaesthesiologists. THR=total hip replacement. MoM=metal-on-metal. MoP=metal-on-polyethylene. CoP=ceramic-on-polyethylene. CoC=ceramic-on-ceramic. CoM=metal-on-ceramic. Undetermined =a combination of unclassifiable components *Adjusted p value<0.05

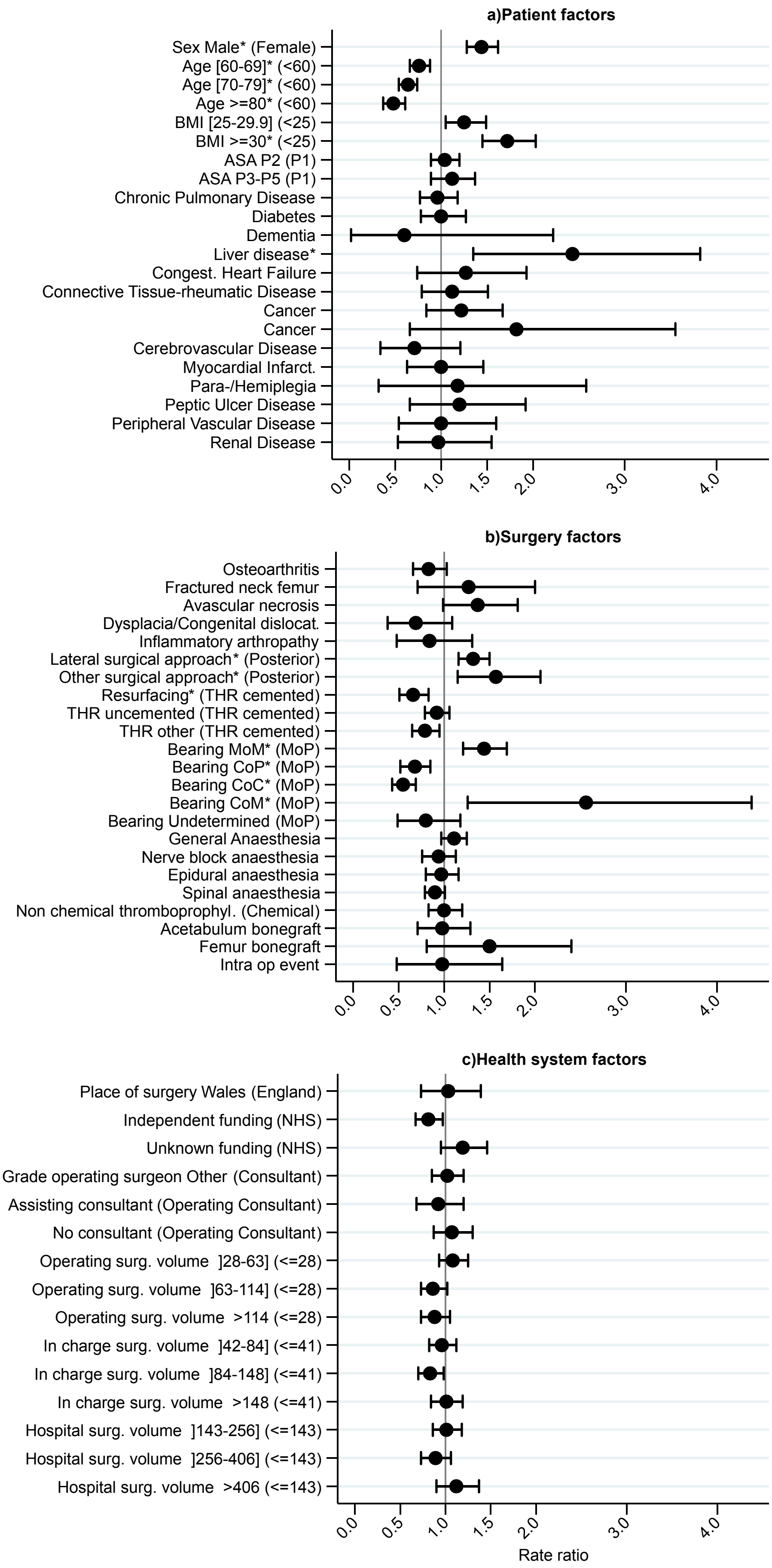

Appendix Figure 4: Risk factors of revision for prosthetic joint infection for the ≥24 postoperative months
Reference category in parentheses. BMI=body-mass index. ASA=American Society of Anaesthesiologists. THR=total hip replacement. MoM=metal-on-metal. MoP=metal-on-polyethylene. CoP=ceramic-on-polyethylene. CoC=ceramic-on-ceramic. CoM=metal-on-ceramic. Undetermined =a combination of unclassifiable components *Adjusted p value<0.05
